# Extracellular vesicle biomarkers in pancreatic ductal adenocarcinoma: from bulk detection to single-extracellular vesicle profiling and endoscopic liquid biopsy

**DOI:** 10.1007/s00535-026-02350-3

**Published:** 2026-02-06

**Authors:** Nana Shimamoto, Kazuki Sumiyama, Yu Fujita

**Affiliations:** 1 Department of Endoscopy, The Jikei University School of Medicine, Minato, Tokyo Japan; 2 Division of Next-Generation Drug Development, Research Center for Medical Sciences, The Jikei University School of Medicine, Minato, Tokyo Japan; 3 Center for Exosome Medical Research, The Jikei University School of Medicine, Minato, Tokyo Japan

**Keywords:** Extracellular vesicles, Liquid biopsy, Pancreatic ductal adenocarcinoma, Single-EV analysis

## Abstract

Pancreatic ductal adenocarcinoma (PDAC) remains a deadly malignancy owing to its late presentation and the limited sensitivity of current serum biomarkers and imaging for early detection. Extracellular vesicles (EVs), which carry proteins, nucleic acids, and lipids that reflect tumor–stromal interactions, have emerged as promising biomarkers for early diagnosis, disease monitoring, and treatment response. Several EV-derived proteins, microRNAs, long noncoding RNAs, and DNA alterations linked to early carcinogenesis and therapeutic resistance have been identified. However, variability in pre-analytical handling and analytical platforms has hindered reproducibility. Global standardization efforts, such as European Liquid Biopsy Society (ELBS), The Blood Profiling Atlas in Cancer (BloodPAC), International Liquid Biopsy Standardization Alliance (ILSA), and Minimal information for studies of extracellular vesicles (MISEV), are currently helping to unify methodologies for EV isolation and molecular profiling. Advances in analytical technologies have shifted the field from bulk EV measurements to high-resolution single-vesicle approaches. Techniques, such as nanoflow cytometry, super-resolution imaging, Raman spectroscopy, and surface-enhanced Raman scattering, enable the detection of rare, mutation-bearing, or functionally distinct EV subpopulations, which may enhance diagnostic precision. In gastroenterology, a major opportunity lies in integrating single-EV analytics with the endoscopic sampling of tumor-proximal fluids. EVs obtained from pancreatic juice, bile, duodenal fluid, or portal venous blood via endoscopic retrograde cholangiopancreatography (ERCP) or endoscopic ultrasound (EUS) provide spatially enriched molecular information beyond peripheral blood. Combining endoscopic access with particle-level EV characterization may allow real-time, mechanism-informed assessment of tumor biology and premalignant lesions, offering a promising strategy for early detection and risk stratification in PDAC. Together, these developments have positioned EV-based liquid biopsy as a rapidly maturing field with strong translational potential.

## Overview of pancreatic ductal adenocarcinoma detection

Pancreatic ductal adenocarcinoma (PDAC) is one of the most lethal malignancies, with an overall 5-year survival rate of approximately 10% and more than 500,000 new cases annually worldwide [1]. Mortality rates are rising globally and correlate strongly with the socio-demographic index (SDI), particularly in middle-SDI regions where future increases are anticipated [1, 2]. Disability-adjusted life-years have nearly doubled over the past two decades [2], underscoring the global need for improved early detection strategies.

Identifying high-risk individuals and implementing structured surveillance are key to early diagnosis. Established risk factors include germline mutations [3], aging [4, 5], race [5, 6], chronic pancreatitis [7, 8], new-onset diabetes mellitus (NOD) [9], and viral hepatitis [10]. Surveillance strategies vary across regions: U.S. guidelines recommend annual surveillance for carriers of high-risk germline mutations [11], whereas European criteria require additional familial risk [12]. In Japan, surveillance focuses on individuals with familial PDAC, with particular emphasis on NOD, chronic pancreatitis, and intraductal papillary mucinous neoplasm as diagnostic entry points [13].

Despite increasing recognition of PDAC risk factors, early detection in population-based screening remains limited. This is largely due to the absence of disease-specific biomarkers and the intrinsic limitations of imaging. CA19-9, the most widely used biomarker, has a sensitivity of only ~ 50% in resectable disease [14, 15], while DUPAN-2 shows similarly insufficient performance [16]. Both are influenced by fucosyltransferase (FUT2/3) genotypes, which substantially affect baseline serum levels and limit their utility for early stage screening [17–19].

Therefore, imaging plays a central role in PDAC detection, with modalities used in a complementary, stepwise manner. Abdominal ultrasound (AUS) is commonly used as a first-line tool but is operator dependent and limited by body habitus, yielding sensitivities of approximately 64–67% [20]. Contrast-enhanced computed tomography (CT) serves as a second-line modality with high overall sensitivity of 90% [21], although hypovascular early stage tumors are frequently missed. In tumors < 2 cm, indirect findings, such as main pancreatic duct dilation [22], focal atrophy [23], and fatty infiltration [24, 25], substantially improve detection. For lesions that remain equivocal, magnetic resonance imaging (MRI) and endoscopic ultrasound (EUS) provide higher spatial resolution. MRI with diffusion-weighted imaging achieves sensitivities of 96–98% for small lesions, while MRCP is valuable for detecting subtle ductal abnormalities [26–28]. EUS offers the highest spatial resolution, with sensitivities of 93–95% for small PDACs [29]. Contrast-enhanced harmonic EUS (CH-EUS) further improves lesion conspicuity by enhancing visualization of tumor microvascular patterns. Prospective studies have demonstrated excellent diagnostic accuracy and interobserver agreement for detecting small (≤ 2 cm) PDACs, as well as improved targeting during EUS-guided fine-needle aspiration (EUS-FNA) [30]. In addition, microbubble-based contrast agents exhibit unique biophysical properties, such as sonoporation and cavitation, forming the basis for ultrasound-targeted microbubble destruction (UTMD), an emerging strategy under investigation for enhanced drug delivery and theranostic applications [31–33]. Collectively, these advances highlight the complementary roles of contemporary imaging modalities in improving early PDAC detection. However, imaging alone remains insufficient for population-level screening or for the reliable identification of sub-centimeter lesions, underscoring the need for complementary liquid biopsy–based approaches. Importantly, the high sensitivities reported for advanced imaging techniques—particularly MRI and EUS—largely reflect performance in expert centers or selected cohorts and should not be directly extrapolated to population-based screening.

## Progress of liquid biopsy for PDAC diagnosis

Liquid biopsy has emerged as a promising strategy for the early detection of PDAC, where conventional tumor markers and imaging frequently fail. Among blood-based biomarkers, circulating tumor DNA (ctDNA) directly reflects tumor-derived genomic alterations. Multi-gene methylation panels combined with CA19-9 have achieved high sensitivity and specificity for stage I PDAC, and integrative ctDNA profiling incorporating methylation, fragmentomics, and copy-number variation has demonstrated near-perfect diagnostic performance [34, 35]. In contrast, detection of KRAS mutations alone—despite their presence in nearly 90% of PDACs—remains insufficiently sensitive in early stage disease because of low ctDNA shedding [36], although dynamic changes in KRAS allele burden during treatment have strong prognostic value [37, 38].

Circulating microRNAs (miRNAs) have also been extensively investigated. Meta-analyses indicate that miR-21 and miR-155 show reproducible but moderate diagnostic accuracy, which can be improved by multi-miRNA signatures, particularly when combined with CA19-9　[39]. Retrospective cohort studies further suggest that specific miRNA profiles may precede PDAC diagnosis by several years, although prospective validation is still required [40].

Protein biomarkers remain centered on CA19-9, which is primarily used for disease monitoring and is susceptible to inflammatory confounders [41–43]. Combining CA19-9 with additional proteins has improved diagnostic performance; for example, thrombospondin-2 (THBS2) enhanced accuracy in multicenter studies [44], and multi-analyte panels such as the CA19-9・TIMP1・LRG1 + metabolite panel and the IMMray PanCan-d algorithm showed high accuracy in high-risk cohorts, though population-level screening remains challenging [45–48].

Beyond blood, alternative biofluids offer complementary information. The urinary PancRISK panel demonstrated strong performance for early stage disease and is undergoing prospective validation [49, 50], while saliva-based assays detecting tumor-derived RNA species, including miRNA panels, have shown encouraging results but largely remain at the case–control stage [51–56].

Together, these advances highlight substantial progress in non-EV liquid biopsy approaches for PDAC. However, most modalities are limited by low tumor fraction in early stage disease, non-specificity of background signals from benign conditions, and loss of spatial and cellular origin information. In addition, many assays have been evaluated mainly in retrospective or enriched cohorts, limiting their generalizability to true population-based screening. These constraints underscore the need for complementary biomarker platforms that preserve biological context and more directly capture tumor-derived signals—features that are difficult to achieve with free-circulating analytes but are inherently addressed by extracellular vesicles.

## The potential of EV in PDAC

### Fundamental EV biology

Extracellular vesicles (EVs) are membrane-bound nanoparticles released by most cell types and function as mediators of intercellular communication. EVs consist of a lipid bilayer enclosing bioactive cargo, including proteins, RNAs, DNA, and lipids, which protects these molecules from extracellular degradation.

**Fig. 1:**
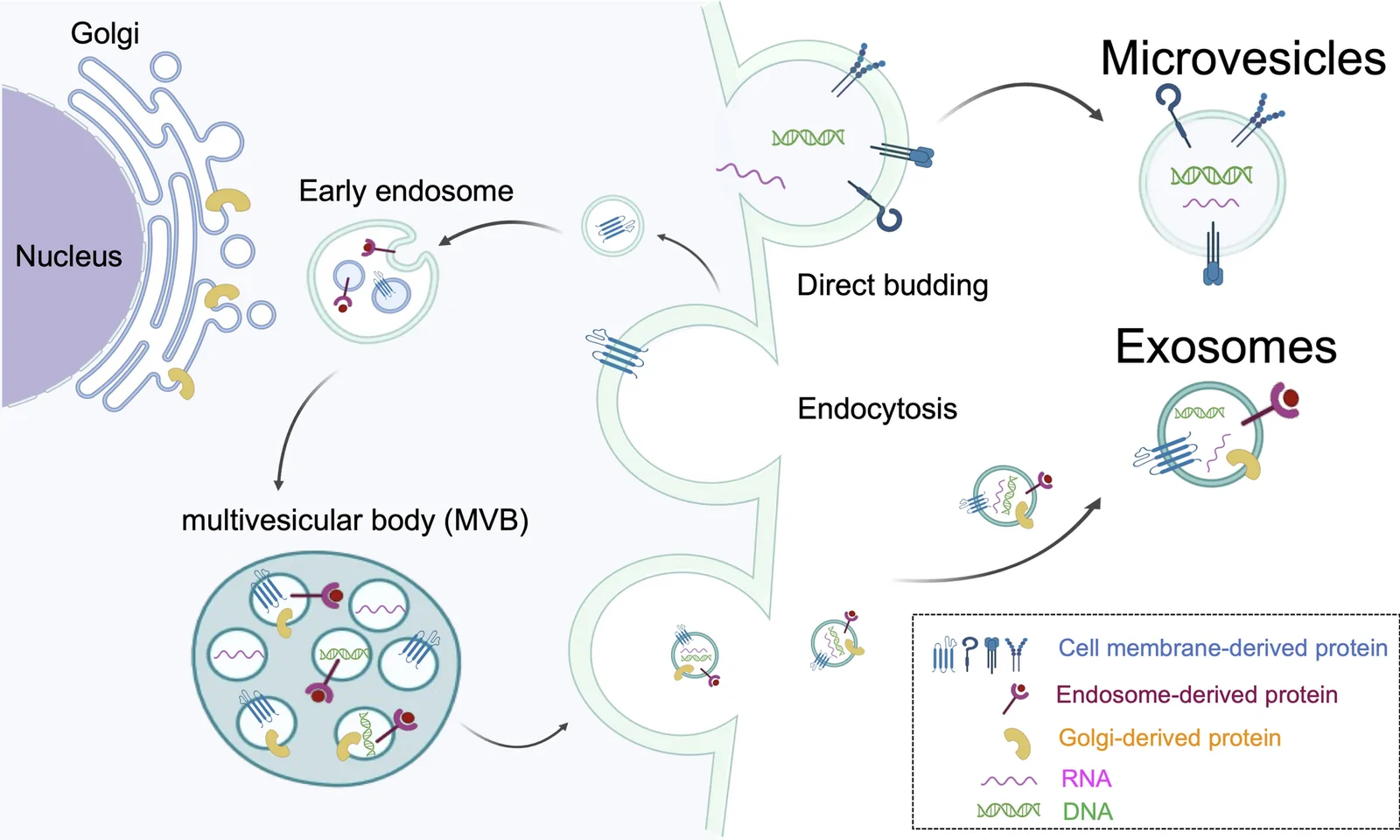
Fundamental EV Biology. Extracellular vesicles (EVs) are broadly classified into exosomes, generated by endocytosis and through the endosomal multivesicular body (MVB) pathway, and microvesicles, which arise from direct budding of the plasma membrane. Exosomes selectively incorporate membrane proteins derived from the cell membrane, endosomal system, and Golgi apparatus, as well as RNAs and DNA actively sorted into intraluminal vesicles, thereby reflecting regulated cellular signaling states. In contrast, microvesicles capture membrane components and nearby cytosolic proteins, RNAs, and DNA at the time of membrane budding, representing a snapshot of the local cellular environment with lower cargo selectivity

EVs are broadly categorized by their biogenesis into exosomes, which originate from the endosomal multivesicular body (MVB) pathway, and microvesicles, which form by direct budding from the plasma membrane [57]. Exosomes selectively incorporate membrane proteins derived from multiple intracellular compartments, including the plasma membrane, endosomal membranes, and the Golgi apparatus, reflecting regulated trafficking through the endosomal pathway. Their luminal cargo—such as RNAs and DNA fragments—is also selectively loaded through active sorting mechanisms involving RNA-binding proteins and endosomal machinery, thereby encoding specific signaling states of the originating cells. In contrast, microvesicles capture membrane components and nearby cytosolic contents at the time of plasma membrane budding. As a result, their cargo composition more closely represents a snapshot of the local cellular environment, including adjacent proteins, RNAs, and DNA, with comparatively lower selectivity than exosomes. Together, these distinct biogenetic mechanisms confer fundamental differences in cargo composition and biological information content between exosomes and microvesicles, providing a conceptual basis for interpreting EV heterogeneity and their differential utility as liquid biopsy biomarkers [58] (Fig. 1).

#### Biogenesis & release

In PDAC, EV formation is predominantly driven by the ESCRT/MVB pathway, with Raba-regulated secretion playing a functional role in EV release and metastatic niche formation [59, 60]. Organotropism is mediated by EV surface integrins: αvβ5 directs EVs to hepatic Kupffer cells to prime liver premetastatic niches [61]. A landmark study further showed that MIF-enriched PDAC EVs activate Kupffer cells, generating TGF-β/IL-10-rich microenvironments that recruit immunosuppressive myeloid cells [62]. Cargo sorting is critically shaped by SUMOylated RNA-binding proteins (RBPs), such as hnRNPA2B1, YBX1, and SYNCRIP [63–65]. Importantly, *KRASG12D* enhances SUMOylation via SAE1, strengthening hnRNPA1–TSG101 interactions and loading prometastatic RNAs that promote lymphangiogenesis [66]. Microenvironmental stress further alters cargo composition; under hypoxia, EVs are enriched in miR-301a-3p, which drives macrophage M2 polarization and metastasis [67]. These mechanistic studies highlight how oncogenic signaling and environmental stress dynamically reprogram the EV content to support tumor progression.

#### Uptake & organotropism

EV internalization occurs through lipid raft-dependent and dynamin-mediated endocytosis [68], but macropinocytosis (MP) is particularly relevant in PDAC, since *KRAS* mutations occur in approximately 90% tumors. MP serve as a major uptake route [53, 69–72] and form the basis of the iExosome KRASG12D-siRNA therapeutic platform, which exploits CD47-mediated immune evasion and MP uptake for potent in vivo tumor suppression　[72]. Heparan sulfate proteoglycans (HSPGs) shape EV binding, and GPC1 functions as a key EV receptor in PDAC [73, 74]. In macrophages and hepatocytes, TIM4 binds to phosphatidylserine (PS) and mediates EV clearance and functional uptake [62, 68]. At the systemic level, integrin-defined organotropism directs PDAC EVs predominantly to the liver [61], and highly metastatic PDAC lines secrete EVs with stronger hepatic tropism, linking EV biology to metastatic behavior [75]. Taken together, fundamental EV biology in PDAC reveals a unifying principle: extracellular vesicles are not passive byproducts of tumor growth, but actively programmed communication units that integrate oncogenic signaling, microenvironmental stress, and tissue-specific targeting. Through coordinated regulation of biogenesis, cargo selection, and cellular uptake, PDAC-derived EVs transmit biologically meaningful information that shapes immune modulation, metastatic behavior, and organotropism. This systems-level view provides the conceptual framework for understanding why EVs are not only central to PDAC pathobiology but also uniquely suited as tumor-proximal and mechanistically anchored biomarkers.

### EV-mediated malignant programming in pancreatic cancer

#### Tumor–stroma crosstalk

EVs orchestrate multiple facets of tumor progression. PDAC EVs activate pancreatic stellate cells into myofibroblast-like CAFs, increasing α-SMA and collagen I expression within hours [76]. EV-mediated angiogenesis is context-dependent; Rab27a-driven EVs suppress angiogenesis [77], whereas EVs enriched in hypoxia-induced miRNAs, such as miR-30b-5p, miR-210, and miR-27a, promote vascular remodeling [78–80]. Immunomodulation is an important mechanism. miR-301a-3p EVs reprogram macrophages toward the M2 phenotype via PTEN/PI3Kγ signaling [67]. Further studies have shown that CAF- or cancer-derived EV cargos, including miR-210 [79], miR-510 [81], *KRASG12D*-EV-activating STAT3 [82], and CAF-EV miR-320a [83], collectively form an immunosuppressive microenvironment. EV-mediated metabolic crosstalk between tumors and pancreatic islets also contributes to PDAC-associated NOD: EV-carried adrenomedullin (ADM) induces ER stress and impairs GSIS [84], while miR-19a-containing EVs disrupt β-cell differentiation and insulin secretion [85, 86].

#### Immune modulation & drug resistance

PDAC is a prototypical “cold tumor.” PDAC-derived EVs deliver PD-L1, TGF-β, and FasL, suppressing dendritic cells and NK cells and inducing T-cell exhaustion [87]. In the liver, EV-MIF signaling primes Kupffer cells to release TGF-β/IL-10 and recruit M2-like macrophages while increasing PD-L1 expression (61, 62, 88). Chemoresistance is strongly EV-driven. Tumor-derived EVs downregulate DCK and enhance antioxidant pathways [89], thereby promoting primary gemcitabine resistance. Hypoxia-induced or drug-exposed PDAC cells release EVs that augment glycolysis, glutamine metabolism, fatty acid oxidation, and autophagy [90]. EV cargos, such as miR-155 [91], MMP14-positive EVs [92], and miR-210 [93], horizontally propagate resistant phenotypes and accelerate secondary resistance throughout the tumor ecosystem. Taken together, these findings illustrate that PDAC-derived EVs dynamically encode oncogenic signaling, stromal remodeling, immune evasion, and metabolic reprogramming. This mechanistic diversity is directly reflected in the molecular cargo of the circulating EVs, providing a strong biological rationale for exploiting EV-associated proteins, RNAs, and DNA as integrated biomarkers for PDAC detection and monitoring (Table 1). Collectively, EV-mediated malignant programming in PDAC reflects a coordinated communication system through which tumor cells reshape stromal architecture, suppress antitumor immunity, and disseminate therapy-resistant traits. Rather than acting via isolated mechanisms, PDAC-derived EVs integrate oncogenic, metabolic, and immune signals to reprogram diverse recipient cells across the tumor microenvironment. This integrated reprogramming is directly reflected in the molecular cargo of circulating EVs, providing a biologically grounded link between tumor behavior and liquid biopsy readouts and supporting the use of EV-associated analytes for PDAC detection and disease monitoring.

**Table 1:** EV-mediated malignant programming in pancreatic cancer

Subsection	Origin cell type	Key molecules	Mechanistic summary (pathway format)	Refs.
Tumor–stroma crosstalk	PSCs/PDAC	let-7 family miRNAs	acinar-EV let-7 s ↓ → PSC activation ↓ → fibrosis attenuation	[75]
	PDAC	miR-30b-5p, miR-210, miR-27a	hypoxia → EV-miRNAs ↑ → GJA1/BTG2/EFNA3 suppression → angiogenesis ↑	[76–79]
	PDAC/CAF	miR-301a-3p, miR-210, miR-510, miR-320a, KRAS^G12D	EV cargos → PTEN/PI3Kγ or STAT3 modulation → macrophage M2 polarization → immunosuppression	[66, 78–82]
	PDAC	ADM, miR-19a	tumor EVs → ADM/miR-19a transfer → β-cell GSIS ↓ & insulin production ↓ → paraneoplastic diabetes	[83–85]
Immune modulation & drug resistance	PDAC-	PD-L1, TGF-β, FasL	EV PD-L1/TGF-β/FasL → DC & NK suppression → T-cell exhaustion → immune evasion	[86]
	PDAC	MIF	PDAC-EV MIF → Kupffer cell activation → TGF-β/IL-10 ↑ → M2 recruitment → hepatic immunosuppressive niche	[60, 61, 87]
	PDAC	miR-155	EV miR-155 → DCK suppression → ROS detox ↑ → gemcitabine resistance	[88]
	PDAC	Metabolic cargos	hypoxia/drug stress → EV-mediated glycolysis/FAO/autophagy ↑ → survival under therapy	[89]
	PDAC	miR-155, MMP14, miR-210	EV cargos → CD44 stabilization/survival pathways ↑/sensitizing genes ↓ → resistance transmission	[90–92]

### Current progress in EV-derived biomarkers for PDAC

Extracellular vesicle (EV)-based biomarkers represent a distinct class of liquid biopsy analytes that complement established circulating biomarkers, such as soluble protein markers, circulating tumor DNA (cfDNA), and circulating free RNA (cfRNA). Compared with freely circulating molecules, EVs offer several conceptual advantages, including protection of molecular cargo from enzymatic degradation, enrichment of tumor-derived signals through selective cargo loading, and partial preservation of cellular origin information. These features are particularly attractive for early stage pancreatic ductal adenocarcinoma (PDAC), where tumor-derived signals are scarce.

At the same time, EV-based approaches present important practical limitations relative to other liquid biopsy modalities. EV isolation and molecular profiling require additional analytical steps, increasing technical complexity, cost, and susceptibility to pre-analytical and inter-laboratory variability. In contrast, cfDNA- and cfRNA-based assays benefit from simpler workflows and more advanced standardization, while soluble protein markers remain highly accessible and scalable despite limited disease specificity. Moreover, the lack of universally standardized EV isolation, quantification, and normalization protocols remains a major barrier to direct comparison across studies and to widespread clinical implementation.

Against this background, EV-derived biomarkers for PDAC have evolved along four major axes—surface proteins, encapsulated miRNAs, long noncoding RNAs, and exosomal DNA—each offering distinct strengths and limitations. The following sections summarize the current progress in these categories, with particular attention to their clinical purpose, analytical feasibility, and translational challenges.

#### EV proteins

EV surface proteins are primarily investigated as diagnostic and prognostic biomarkers, leveraging their accessibility to antibody-based detection platforms. Their major strengths include direct cell-of-origin information and compatibility with established assays such as ELISA. However, diagnostic performance is highly sensitive to pre-analytical handling, EV isolation methods, and antibody specificity, resulting in variable reproducibility across studies.

Among the surface proteins, glypican-1 (GPC-1) is one of the earliest and most widely discussed EV markers for PDAC. Melo et al*.* first reported that GPC1-positive EVs can diagnose PDAC with remarkable accuracy across all stages, achieving 100% sensitivity and 100% specificity even for stage I disease [94]. Subsequent meta-analyses also supported its potential diagnostic value, with pooled sensitivity, specificity, and AUC values of 0.88, 0.86, and 0.93, respectively [95]. However, follow-up studies have yielded inconsistent results, with some failing to demonstrate statistical significance using ELISA [96] or reporting low diagnostic accuracy (reduced AUC) [97], raising concerns about reproducibility. Such variability is likely attributable to differences in cohort composition, pre-analytical handling, EV isolation and enrichment protocols, and detection methods among studies [98].

Combined approaches have been explored to overcome these limitations. Integrating CD82 and CA19-9 with GPC-1 improved the diagnostic performance (AUC = 0.942) [99], and a composite index that quantified GPC-1 EV mRNA levels achieved an AUC of 0.947 for stage I PDAC [100]. These findings suggest that combining GPC-1 with other molecular markers may enhance the diagnostic robustness beyond single-analyte performance.

EphA2, a member of the EPH receptor tyrosine kinase family, is a promising EV-associated biomarker. Its overexpression and aberrant non-canonical activation have been implicated in tumor invasion, metastasis, and malignant progression [101]. An ELISA-based analysis of 353 samples (including samples from 204 patients with PDAC) reported that exosomal EphA2 levels were significantly elevated in PDAC, and when combined with CA19-9 or CA242, improved discrimination between early stage (I/II) PDAC, benign pancreatic disease, and healthy individuals [102].

High exosomal EphA2 levels are also correlated with poor clinical outcomes. In a cohort of 200 samples (90 patients with PDAC), elevated serum Exo-EphA2 levels were strongly associated with shorter overall survival (OS) and increased recurrence risk, remaining an independent prognostic factor in multivariate analysis. Notably, baseline Exo-EphA2 levels at initial diagnosis correlated with values at recurrence, implying a link between tumor relapse and EV-mediated EphA2 signaling [103].

Moreover, Exo-EphA2 has been implicated in chemoresistance. In experimental models, exosomes derived from gemcitabine-resistant PDAC cells transferred drug resistance to their sensitive counterparts, which was abrogated by EphA2 knockdown in donor cells [104]. These findings suggested that Exo-EphA2 acts as a functional mediator of chemotherapeutic resistance in PDAC.

#### EV miRNA

EV-associated miRNAs are mainly explored for early detection and disease classification, owing to their relative stability and quantitative detectability. While panel-based approaches improve sensitivity by capturing composite tumor and stromal signals, key limitations include heterogeneity in RNA extraction methods, normalization strategies, and analytical pipelines, which complicate cross-study standardization and reproducibility.

Among EV-associated miRNAs, miR-21 is the most extensively studied, with 53–87% sensitivity and 82–100% specificity [97]. Other frequently reported diagnostic candidates for PDAC include miR-451a (66–80% sensitivity, 86–90% specificity) and miR-10b (42–100% sensitivity 90–100% specificity). However, these individual markers often exhibit variability in sensitivity, raising concerns about their standalone diagnostic performance.

In this context, there has been a notable shift toward diagnostic panels that combine multiple miRNAs. In PDAC, cancer cells and components of the surrounding microenvironment secrete EVs with distinct miRNA profiles. Consequently, the miRNA landscape within circulating EVs reflects an integrated transcriptional “fingerprint” of both tumor and stromal compartments [105]. Panel-based diagnostics aim to capture this composite EV miRNA signature, not merely as an additive combination of multiple markers but also as a holistic readout of the tumor microenvironment.

To date, more than 100 diagnostic panels have been developed. Meta-analyses demonstrated pooled sensitivity and specificity of approximately 80% and 88%, respectively, for PDAC diagnosis using miRNA panels [97]. Moreover, some studies suggest that these panels outperform GPC-1 alone in differentiating PDAC from chronic pancreatitis [106]. This is particularly significant, since chronic pancreatitis, a condition involving inflammation, fibrosis, and premalignant changes, can present blood biomarker profiles similar to those of PDAC. Since various EVs are secreted by not only tumor cells, but also stromal and immune cells, multi-miRNA panels may serve as integrative signatures that better capture PDAC against the backdrop of chronic inflammation. Furthermore, a few studies have combined EV-derived miRNAs with cell-free RNA (cfRNA). One such report showed that a diagnostic model combining eight EV-miRNAs and five cfRNAs from serum achieved an AUC of 0.93 for early stage PDAC (stage I/II), and 0.96 even in CA19-9-negative patients [107]. Exosomal miRNAs predominantly reflect active, regulated secretion from viable cells, and thus specific pathway activities, whereas cell-free miRNAs are passively released via necrosis, apoptosis, or leakage into the circulation, capturing a broader and less selective input from tumor, stromal, and immune components. Thus, combining exosomal and cfRNA-derived miRNA signatures provides a more comprehensive view of the transcriptomic landscape, encompassing both active and passive regulatory layers, which is a novel dimension for PDAC diagnosis.

#### EV-lncRNA

EV-derived lncRNAs are emerging as candidate biomarkers for tumor specificity and molecular subtyping, reflecting higher order transcriptional and epigenetic regulation. Their strengths lie in potential tumor specificity and functional relevance, but clinical translation is limited by low abundance, platform-dependent detection variability, and a current lack of standardized analytical frameworks.

Xie et al. analyzed salivary EV-derived lncRNAs from 55 patients with PDAC, 55 healthy individuals, and 20 individuals with benign pancreatic disease and identified HOTAIR and PVT1 as potential diagnostic markers. The diagnostic performance for distinguishing PDAC from healthy individuals showed sensitivities and specificities of 78.2% and 85.6% for HOTAIR, and 96.4% and 63.6% for PVT1, respectively; when combined, the model achieved 88.2% sensitivity and 90.9% specificity [108]. Kumar et al. comprehensively analyzed the RNA cargo within serum-derived EVs from patients with PDAC, IPMN, and healthy individuals. They identified the differential expression of CRNDE and MALAT-1, both of which were significantly elevated in patients with PDAC than in healthy individuals [109]. Although statistical measures, such as AUC, have not been reported, this study demonstrated that EV-associated lncRNAs can reflect tumor histotypes, supporting their potential as diagnostic targets within the EV-RNA profile. Takahashi et al. have reported the diagnostic and functional significance of HULC, which discriminated PDAC from non-malignant groups in serum samples from 20 patients with PDAC, 22 patients with IPMN, and 21 healthy individuals with 80% sensitivity, 92.1% specificity, and an AUC of 0.92. Functionally, HULC expression was induced by TGF-β1 and promoted epithelial–mesenchymal transition (EMT), a process regulated by miR-133b [110]. Panel-based approaches that combine multiple lncRNAs have also been proposed. A four-lncRNA panel (LINC01268, LINC02802, AC124854.1, and AL132657.1) showed diagnostic sensitivity, specificity, and AUC values of 0.72, 0.89, and 0.848, respectively, for PDAC [111]. Emerging evidence has revealed functional roles of EV-derived lncRNAs in PDAC pathophysiology. For instance, the lncRNAs UCA1 [112], SNHG11 [113], and LINC00460 [114] enhance malignant phenotypes through miR-96-5p, miR-324-3p, and miR-503-5p, respectively; lncRNA-Sox2ot downregulation promotes metastasis [115] and lncROR contributes to gemcitabine resistance via regulating the Hippo signaling pathway [116]. Although these findings highlight the multifaceted functions of EV-lncRNAs in PDAC, their clinical utility as diagnostic, prognostic, and predictive biomarkers remains at an early stage of development. Further studies are warranted to determine whether these molecules can serve as indicators of the prognosis, therapeutic response, and drug resistance in patients with PDAC.

#### EV-DNA

EV-associated DNA is primarily investigated for mutation detection and treatment response monitoring, offering enhanced protection and tumor specificity compared with cell-free DNA. Although EV-DNA shows promise for early stage detection and prognostication, challenges remain in harmonizing isolation protocols, distinguishing EV-bound from contaminating DNA, and establishing standardized quantitative thresholds.

Kahlert et al. first demonstrated that tumor-derived double-stranded DNA (dsDNA) was encapsulated within EVs by analyzing *KRAS* and *TP53* mutations in EVs isolated from PDAC cell lines and patient serum. They confirmed that PDAC-derived EV-DNA contained mutated *KRAS* and *TP53* sequences [117]. Subsequent studies revealed that circulating EVs from patients with PDAC allow for the rapid and cost-effective detection of *KRAS* and *TP53* mutations, underscoring the feasibility of EV-DNA as a driver mutation detection tool [118]. Comparative analyses between EV-DNA and cell-free DNA (cfDNA) across disease stages further showed that the detection rate of mutant *KRAS* in EV-DNA increased with disease progression and that EV-DNA demonstrated higher sensitivity in early stage PDAC [119]. These findings suggest that EV-DNA is more resistant to degradation and exhibits higher tumor specificity than cfDNA, which often contains a higher background of DNA derived from normal cells. Building on this concept, Bernard et al. conducted a large-scale comparative study of exoDNA and circulating tumor DNA (ctDNA) in 194 patients with localized or metastatic PDAC. They found that exoDNA showed higher detection sensitivity and concordance with tumor tissue (95.5% vs 68.2%), and that patients with *KRAS* mutant allele frequency (MAF) ≥ 5% had significantly shorter progression-free survival (PFS) and OS. Moreover, an increase in exoDNA KRAS MAF (≥ 1%) during therapy preceded radiological progression by a median of 50 days, outperforming CA19-9 and ctDNA in the early prediction of treatment failure [120]. Collectively, these findings highlight exoDNA as a promising prognostic and treatment response monitoring tool for PDAC, complementing and potentially surpassing existing cfDNA-based liquid biopsy approaches in selected settings.

#### Multi-omics

Previous studies on EV biomarkers have largely focused on individual molecular layers and examined EV-derived RNA (miRNAs and lncRNAs), proteins, or DNA in isolation. Recently, however, there has been a growing trend toward multi-omics and integrative approaches in which these diverse molecular hierarchies within EVs are analyzed simultaneously and modeled using AI-based machine learning frameworks. Hinestrosa et al. analyzed the plasma EV protein profiles of 105 patients with early stage PDAC and 545 healthy individuals using the *Exovita* platform. They then integrated these proteomic data with clinical variables, such as age, sex, and CA19-9, through LASSO and logistic regression-based machine learning to construct a multivariate classifier, referred to as an “EV classifier.” This integrated model achieved remarkably high diagnostic performance for early PDAC, with a sensitivity of 93%, specificity of 91%, and AUC of 0.97, clearly outperforming CA19-9 [121]. Similarly, Bockorny et al. performed LC–MS-based proteomic profiling of serum EVs from 124 patients, including 93 with PDAC. By combining bioinformatics integration, encompassing statistical, network, transcriptional, and clinical dimensions, they established a multi-layered analytical framework linking the EV proteome, tumor gene expression, and clinical outcomes. Their results demonstrated a robust predictive performance: PDCD6IP, SERPINA12, and RUVBL2 achieved an AUC of 0.89 for PDAC diagnosis; PSMB4, RUVBL2, and ANKAR predicted metastasis with an AUC of 0.86; and CRP, RALB, and CD55 predicted poor prognosis with an AUC of 0.83 [122]. Since EVs inherently encapsulate heterogeneous molecular cargo across multiple biomolecular layers, they offer an ideal platform for multiparametric multi-omics modeling and AI-driven optimization. Such integrative strategies hold great promise for biomarker discovery and precise PDAC classification. Although these approaches remain at the research stage, continued progress in EV assay standardization and multi-institutional prospective validation are expected to accelerate their clinical implementation soon (Table 2).

**Table 2:** Current progress in EV-derived biomarkers for pancreatic cancer

Category	EV marker(s)	Sample	Cohort (PDAC stage included)	Diagnostic performance (SEN/SPE/AUC)	Refs
protein	GPC1	Serum	PDAC 190 (Stage NA), benign 26, healthy 100	100%/100%/1.0	[93]
protein	EphA2	Serum	PDAC 204 (Stage I–II: 62; III–IV: 142), benign 75, healthy 74	93%/89%/0.94	[101]
protein	CA15-3, CA19-9, Ferritin, FGF-2, sHER3, Leptin, Prolactin	Plasma	Early PDAC 105 (Stage I: 35; Stage II: 70) vs controls 545 (Validation: 30 vs 83)	90–93%/91–93%/0.97	[120]
miRNA	miRNA panel (miR-10b, miR-21, miR-30c, etc.)	Serum/plasma	PDAC 29 (Stage NA) vs healthy 6	80–90%/85–95%/0.93	[105]
miRNA + cfRNA	8-miRNA + 5-cfRNA	Plasma	Early PDAC 44 (Stage I–II) vs controls 57 (Validation total n = 191)	90.9%/91.2%/0.94	[106]
lncRNA	HOTAIR + PVT1	Saliva	PDAC 55 (Stage NA), benign 20, healthy 55, other GI 48	~ 80%/~ 90%/NA	[107]
lncRNA	HULC	Serum	PDAC 20 (Stage I–II: 12; III–IV: 8), IPMN 22, healthy 21	85%/83%/0.89	[109]
lncRNA panel	HEVEPA + HULC + CA19-9	Serum	PDAC 20 (Stage I–II: 12; III–IV: 8), IPMN 22, healthy 21	NA/NA/NA (Improved vs CA19-9)	[129]
DNA	dsDNA (KRAS/TP53)	Serum	PDAC 2 (Stage NA) vs healthy 2	NA/NA/NA (Proof-of-concept only)	[116]

Sensitivity and specificity values for salivary EV-HOTAIR were approximated from ROC curve figures, as exact numerical data were not provided in the original publication

Overall, progress in EV-derived biomarkers for PDAC reflects a transition from single-analyte approaches to integrative, biology-informed diagnostic strategies. EV-associated proteins, RNAs, and DNA consistently enrich tumor-derived signals and improve discrimination from inflammatory pancreatic diseases, but the performance of individual markers remains variable and context dependent. In contrast, panel-based and multi-omics approaches exploit the intrinsic heterogeneity of EV cargo to achieve more robust disease classification. Although further standardization and prospective validation are required, mechanistic insights into EV biology are rapidly advancing. The convergence of these insights with integrative analytics positions EV-derived biomarkers as a promising foundation for next-generation liquid biopsy platforms in PDAC.

## Advances in EV isolation, molecular profiling, and data interpretation technologies in PDAC

### Challenges in EV analysis

Despite rapid progress in extracellular vesicle (EV)-based biomarker research, several fundamental challenges continue to limit the clinical translation of EV analyses, particularly in pancreatic ductal adenocarcinoma (PDAC). These challenges primarily involve pre-analytical variability, limited reproducibility, and insufficient standardization.

Pre-analytical variability remains a major source of inconsistency. Factors, such as sample collection, processing conditions, storage, and contamination by abundant serum proteins or lipoproteins, can substantially affect EV yield and cargo composition. In PDAC, where tumor-derived EVs constitute only a small fraction of circulating vesicles, even minor deviations may lead to disproportionate signal distortion and false-positive or false-negative results.

Reproducibility and standardization represent additional barriers. Conventional EV isolation and quantification methods are labor-intensive and highly operator dependent, resulting in inter-laboratory variability and limited quantitative robustness. Moreover, the absence of standardized workflows across EV enrichment, molecular detection, normalization, and data interpretation complicates multicenter validation and regulatory translation.

Together, these limitations underscore the need for next-generation EV technologies that enable simplified, reproducible, and standardized analyses, which are essential for advancing EV-based biomarkers toward routine clinical application in PDAC.

### Isolation and molecular profiling

To address the above-mentioned pre-analytical variability and limited reproducibility, recent advances have focused on simplifying and standardizing EV isolation and molecular profiling workflows. Conventional methods, such as ultracentrifugation and western blotting or ELISA, are labor-intensive and poorly scalable. In contrast, emerging platforms enable rapid, quantitative, and reproducible EV analysis with minimal sample handling, facilitating the clinical translation of EV-based biomarkers. ExoScreen　represents a particularly powerful proximity-based luminescent assay that detects EV directly from crude serum using AlphaLISA bead chemistry. Its wash-free workflow quantifies surface proteins, such as CD9, CD63, and CD147, within 2 h using only 5 μL serum, and its robust analytical sensitivity has been validated using samples from PDAC patient, where ExoScreen successfully distinguished early stage disease from benign tumors [123]. It is particularly attractive for PDAC screening and surveillance, because it requires only microliter-scale serum volumes and minimal sample processing.

The alternating current electrokinetic (ACE) chip employs an ACE microfluidic architecture that concentrates EVs from unprocessed plasma through dielectrophoretic trapping, followed by on-chip immunofluorescent labeling of tumor-associated proteins, including GPC1, EpCAM, and EGFR. This system requires only 30–50 μL blood and provides results within 1 h; importantly, studies in PDAC cohorts have shown that ACE chip-derived EV signatures consistently outperform CA19-9 in diagnostic accuracy and provide better discrimination of early stage disease [124].

Another platform with strong translational potential is EViSTEP®, a technology designed to address two major challenges in EV recovery from biofluids—contamination by impurities and poor reproducibility due to manual handling. Its principle is based on immunoprecipitation (IP) using antibody-conjugated magnetic beads for EV capture. By adding a proprietary pretreatment reagent, the EViSTEP reagent, to the sample effectively removes contaminants from EV surfaces and improves EV recovery efficiency.

Furthermore, the entire EV recovery process is fully automated using a dedicated instrument and completed within 1 h, ensuring high throughput and operational consistency. The utility of this platform has been demonstrated in multiple studies, including the identification of predictive biomarkers for symptomatic pneumonitis during durvalumab consolidation therapy in locally advanced nonsmall cell lung cancer (LA-NSCLC) [125], discovery of poor prognostic factors during the same therapy [126], and development of EV-derived lncRNA biomarkers for PDAC [127]. Together, these applications highlight EViSTEP as a technology with a strong potential for clinical and societal implementation (Table 3).

**Table 3:** Emerging technologies for EV isolation, quantification, and molecular profiling in pancreatic cancer

Technology	Sample volume required	Quantitative performance	Measurement speed/real-time capability	Clinical application/example	Refs.
ExoScreen (AlphaLISA-based proximity assay)	~ 5 μL serum	High quantitative capability; wide dynamic range; detection sensitivity ~ 15 ng EV protein (10^5^–10⁶ EVs)	Not real-time; ~ 2 h per assay	Non-invasive detection of tumor-derived EVs; elevated CD147/CD9 in CRC and early PDAC	[122]
ACE Chip (AC-electrokinetic microfluidic EV isolation)	30–50 μL blood	Quantitative fluorescence-based detection; sensitivity down to 10^3^–10^4^ EVs	Near-real-time (~ 1 h); processing time ~ 1 h	PDAC detection using GPC1, EGFR, EpCAM; improved accuracy vs CA19-9	[126]
EViSTEP (automated immunoprecipitation-based EV recovery)	~ 500 μL (automated workflow)	High reproducibility; low impurity; sensitivity in low-ng EV protein range (10^5^–10^6^ EVs)	Non-real-time; batch processing; ~ 1 h	PDAC detection using EV-lncRNA; NSCLC (durvalumab therapy): prediction of pneumonitis and poor prognosis via proteomics	[127–129]

## Future directions for clinical translation of EV-based PDAC biomarkers

### Global standardization efforts in EV biomarker development

Global standardization is essential for broad implementation of liquid biopsies in clinical practice. To overcome this challenge, international public–private consortia have been established, namely European Liquid Biopsy Society (ELBS) in Europe and BloodPAC in the USA.

ELBS focuses on four key pillars, *technology, education, regulation,* and *data harmonization*, to promote the clinical translation and standardization of liquid biopsy, including EV-based biomarkers [128].

BloodPAC developed the minimal technical data element (MTDE) framework, comprising 11 recommended checklist items to standardize the pre-analytical evaluation of liquid biopsy assays, including those using EVs [129].

These consortia have since converged under the International Liquid Biopsy Standardization Alliance (ILSA), launched within the Foundation for the National Institutes of Health (FNIH) framework. ILSA aims to establish an end-to-end standardized process, from pre-analytical steps and analytical technologies to device validation, clinical sample handling, and data reporting formats, to accelerate regulatory approval and clinical adoption of liquid biopsy assays [130]. In addition, the International Society for Extracellular Vesicles (ISEV) plays a central role in the EV-specific domain, advancing standardization through MISEV guidelines and actively promoting validated protocols for EV isolation, quantification, and data interpretation. While these efforts largely represent *top–down* initiatives focusing on the global harmonization and regulatory alignment of EV-based liquid biopsy technologies, *bottom–up* frameworks are also emerging. In Japan, an industry-driven consortium was established to advance the clinical implementation of EV liquid biopsy for PDAC, integrating the expertise of multiple academic institutions to comprehensively analyze EV-derived miRNAs and proteins. This initiative has accelerated the discovery and clinical translation of EV biomarkers for early PDAC detection, providing a more efficient bridge from research to real-world applications than traditional academia-led studies [131] and may serve as a model for disease-focused EV biomarker consortia in other regions.

### From bulk to single-EV analysis

Single-EV analysis has emerged as a powerful approach for resolving the molecular heterogeneity of EV populations, a feature obscured by conventional bulk assays. Recent technological advances, including high-sensitivity nanoflow cytometry, super-resolution/total internal reflection fluorescence (TIRF) imaging, Raman spectroscopy, and SERS spectroscopy, have enabled the individual analysis to be analyzed individually, allowing not only tumor-specific protein or RNA detection, but also distinct EV subpopulation resolution. By identifying rare tumor-derived vesicles and characterizing their size, surface-marker co-expression, and biochemical signatures, single-EV analysis captures early shifts in EV heterogeneity that are masked in bulk measurements, and therefore is of added diagnostic value for early stage PDAC [132, 133]. Simultaneously, it is important to acknowledge that the majority of single-EV platforms remain at a developmental or research stage. Current methodologies are constrained by high costs, technical complexity, limited throughput, and a lack of standardized analytical workflows, all of which hinder near-term clinical implementation. Consequently, while single-EV analysis offers unprecedented resolution and mechanistic insight, its present utility is primarily confined to exploratory or proof-of-concept studies, and clinical applicability should be interpreted with appropriate caution.

Nanoflow cytometry is a high-sensitivity platform that detects the light scattering and fluorescence signals of nanoscale particles, enabling the absolute quantification of size distribution and particle concentration of 40–200 nm EVs. In addition, its capability for the simultaneous detection of multiple fluorescent markers allows the visualization of EV subpopulations whose structural and molecular heterogeneity is obscured in the conventional bulk analyses. In PDAC, nanoflow cytometry has been used to directly visualize KRAS and TP53 mutant proteins within single-EVs, capturing the heterogeneity of mutation-bearing vesicles and detecting stage I PDAC from plasma [134]. In addition, Yang et al. demonstrated that ALIX-positive EVs are markedly enriched in PDAC and nanoflow cytometry distinguishes whether increased ALIX signals reflect protein upregulation or an expansion of specific EV subpopulations, insights not achievable by ELISA or bulk proteomics [135].

Super-resolution and TIRF imaging are techniques that visualize EV surface information at a resolution of tens of nanometers. Super-resolution microscopy enables the detailed assessment of protein clustering and spatial localization on the EV membrane, detecting structural differences among EV subpopulations. TIRF imaging selectively excites an extremely shallow region near the coverslip surface (approximately 100–200 nm), enabling high signal-to-noise ratio quantification of surface molecule intensity and distribution at the single-EV level. In PDAC, super-resolution imaging of patient-derived plasma EVs revealed the presence of unique EV subpopulations that can only be distinguished through single-EV-level analysis [136].

Raman spectroscopy detects the scattered light arising from molecule-specific vibrational modes, thus providing a unique “chemical fingerprint.” Surface-enhanced Raman scattering (SERS) uses metal nanostructures to amplify these signals, enabling highly sensitive detection of trace molecules and single-EVs. In a previous study, Raman and SERS fingerprinting simultaneously classified six cancer types, including PDAC, with high accuracy, enabling early stage detection based on distinctive lipid and protein vibrational signatures [137]. These developments highlight how high-resolution single-EV analytics are shifting EV biomarker research from bulk quantification to molecularly defined stratification of EV subpopulations with direct diagnostic relevance.

### Targeting liquid biopsy via endoscopy

In addition to conventional liquid biopsy materials, endoscopic approaches provide a unique opportunity for minimally invasive access to pancreatic and biliary fluids, enabling the collection of tumor-proximal samples for liquid biopsy. Pancreatic juice represents a major proximal source of tumor-derived analytes: digital NGS detecting *KRAS*, *SMAD4*, and *GNAS* mutations achieved an AUC of 0.89 for PDAC detection [138]. Although *KRAS* mutations are also observed in precursor lesions, such as PanIN and IPMN, diagnostic yield is highly dependent on sampling timing and technique [139, 140]. Cell-free DNA obtained during ERCP has demonstrated higher variant allele fractions in bile than in plasma [141, 142], and the Bilemut assay applying targeted NGS to bile cfDNA achieved a sensitivity of 96%, correctly identifying malignancies even in cases initially classified as benign or indeterminate [143]. However, distinguishing PDAC from cholangiocarcinoma remains challenging, as bile cfDNA may originate from either tumor site.

Recent studies have further demonstrated that duodenal fluid collected endoscopically, followed by analysis of *KRAS* mutations [144] or EV-derived miRNAs [145], can discriminate early pancreatic cancer, highlighting the potential of this minimally invasive sampling strategy. In addition, portal venous blood obtained via EUS-guided puncture contains higher concentrations of tumor-specific DNA and EVs than peripheral blood, suggesting improved diagnostic sensitivity [146]. Collectively, these approaches using pancreatic juice, bile, duodenal fluid, and portal blood may bridge molecular diagnostics with real-time endoscopic assessment in PDAC.

Despite these advantages, several practical and clinical challenges must be acknowledged before widespread implementation of endoscopy-based liquid biopsy strategies. Endoscopic sampling of pancreatic juice, while offering access to a proximal source of tumor-derived biomarkers, is technically demanding and requires specialized expertise, thereby limiting its scalability and feasibility for routine clinical use [147]. Analyses of bile cfDNA, although promising for cancer detection, are complicated by uncertainty regarding tissue origin, as bile may contain nucleic acids derived from both pancreatic and biliary lesions, potentially confounding diagnostic interpretation [148]. Duodenal fluid-based biomarkers face additional challenges related to dilution effects and pH variability caused by gastric acid exposure, which may degrade analytes and reduce signal fidelity [149]. Importantly, these approaches rely on invasive endoscopic procedures and are therefore unlikely to be suitable for population-based screening. Their clinical utility is more appropriately confined to selected high-risk individuals, patients with indeterminate pancreaticobiliary lesions, or settings in which endoscopic evaluation is already clinically indicated, such as diagnostic work-up or surveillance of high-risk cohorts. Furthermore, broader limitations inherent to liquid biopsy—including variability in sampling conditions and the lack of standardized protocols for cfDNA extraction and analysis—remain significant barriers to reproducibility and widespread adoption [147].

**Fig. 2:**
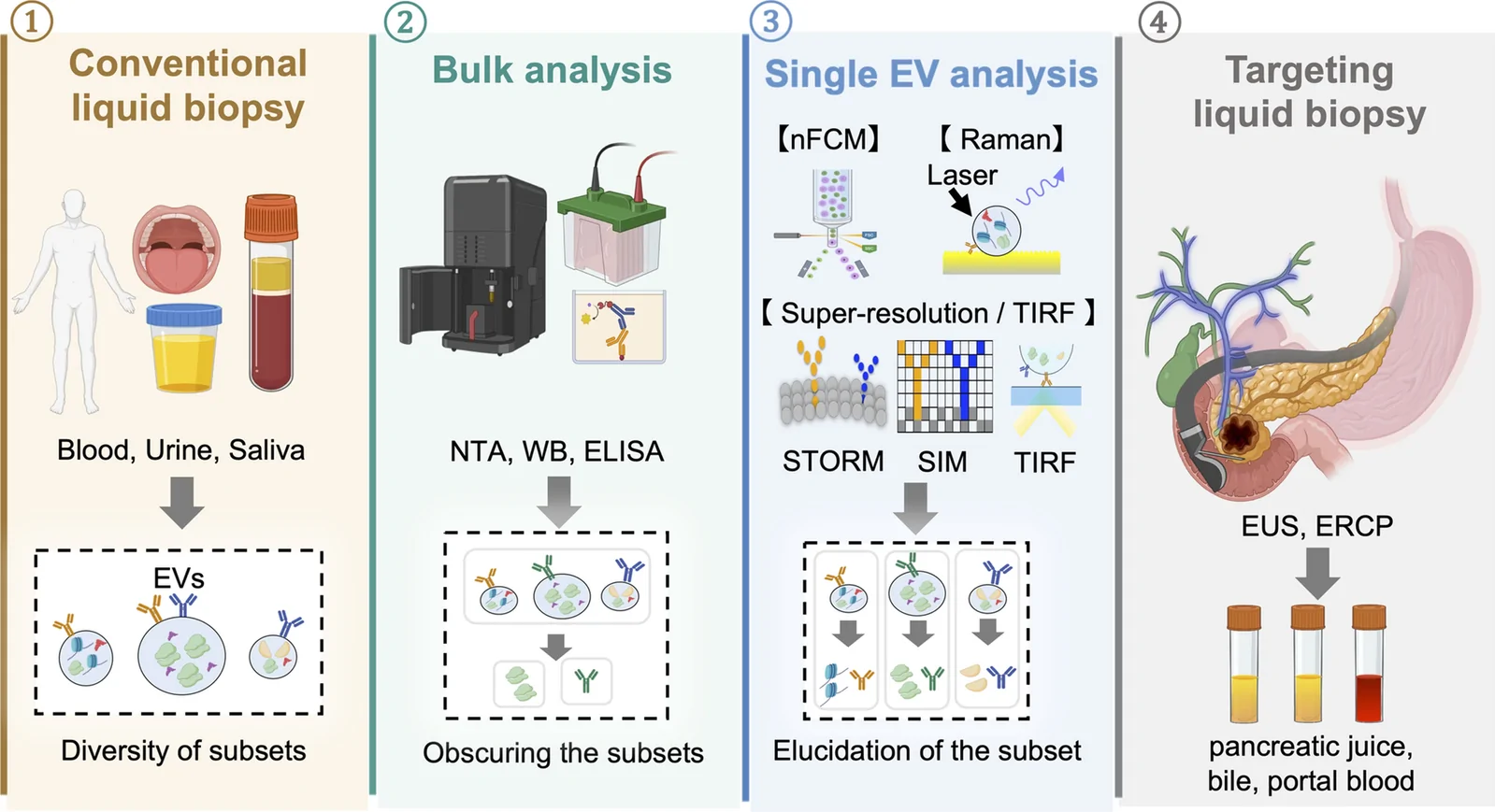
Evolution from conventional bulk extracellular vehicle (EV) analysis toward single-EV analysis and endoscopically targeted liquid biopsy approaches. Conventional liquid biopsy using blood, urine, or saliva captures bulk EVs, which consist of diverse subsets that differ in surface markers and cargo depending on their cellular origin. Bulk EV analysis [Nano particle Tracking Analysis (NTA), Western blotting (WB), and Enzyme-linked immunosorbent assay (ELISA)] quantifies specific markers or cargo but obscures the underlying heterogeneity. In contrast, single-EV platforms [nanoflow cytometry (nFCM), Raman/surface-enhanced Raman scattering (SERS), and super-resolution/total internal reflection fluorescence microscopy (TIRF)] enable the resolution of vesicle-level diversity. Endoscopic approaches [endoscopic ultrasound (EUS)/endoscopic retrograde cholangiopancreatography (ERCP)] further allow the sampling of tumor-proximal fluids, such as pancreatic juice, bile, and portal blood, thereby enhancing the precision of EV-based diagnostics

Nevertheless, endoscopic liquid biopsy offers an unparalleled balance between tumor proximity and clinical feasibility. This approach holds strong promise for enhancing diagnostic sensitivity while preserving biological context through the integration of endoscopic sampling with EV-based molecular analyses. Future standardization efforts and technological refinements will be essential to translate these strengths into safe, reproducible, and clinically actionable strategies for PDAC screening, surveillance, and therapeutic decision-making (Fig. 2).

## Abbreviations

ADM: Adrenomedullin
ACE: Alternating current electrokinetic
AUS: Abdominal ultrasound
BloodPAC: The Blood Profiling Atlas in Cancer
CH-EUS: Contrast-harmonic endoscopic ultrasound
ctDNA: Circulating tumor DNA
CT: Computed tomography
ERCP: Endoscopic retrograde cholangiopancreatography
EUS: Endoscopic ultrasound
EUS-FNA: Endoscopic ultrasound-guided fine-needle aspiration
ELISA: Enzyme-linked immunosorbent assay
ELBS: European Liquid Biopsy Society
EV: Extracellular vesicles
FNIH: Foundation for the National Institutes of Health
GPC-1: Glypican-1
HSPGs: Heparan sulfate proteoglycans
ILSA: International Liquid Biopsy Standardization Alliance
IPMN: Intraductal papillary mucinous neoplasm
ISEV: International Society for Extracellular Vesicles
lncRNAs: Long noncoding RNAs
MP: Macropinocytosis
miRNAs: MicroRNAs
MIDE: Minimal technical data element
MAF: Mutant allele frequency
MRCP: Magnetic resonance cholangiopancreatography
MISEV: Minimal information for studies of extracellular vesicles
NOD: New-onset diabetes mellitus
NTA: Nano particle Tracking Analysis
OS: Overall survival
PDAC: Pancreatic ductal adenocarcinoma
PS: Phosphatidylserine
PFS: Progression-free survival
RBPs: RNA-binding proteins
SDI: Socio-demographic index
STORM: Stochastic optical reconstruction microscopy
SIM: Structured illumination microscopy
SERS: Surface-enhanced Raman scattering
TIRF: Total internal reflection fluorescence microscopy
UC: Ultracentrifugation
UTMD: Ultrasound-targeted microbubble destruction
WB: Western blotting

## References

[1] Filho AM, Laversanne M, Ferlay J, Colombet M, Piñeros M, Znaor A, et al. The GLOBOCAN 2022 cancer estimates: Data sources, methods, and a snapshot of the cancer burden worldwide. Int J Cancer. 2025;156(7):1336–46. 39688499 10.1002/ijc.35278

[2] Pourshams A, Sepanlou SG, Ikuta KS, Bisignano C, Safiri S, Roshandel G, et al. The global, regional, and national burden of pancreatic cancer and its attributable risk factors in 195 countries and territories, 1990–2017: a systematic analysis for the Global Burden of Disease Study 2017. Lancet Gastroenterol Hepatol. 2019;4(12):934–47. 31648972 10.1016/S2468-1253(19)30347-4 PMC7026711

[3] Llach J, Carballal S, Moreira L. Familial pancreatic cancer: current perspectives. Cancer Manag Res. 2020;12:743–58. 32099470 10.2147/CMAR.S172421 PMC6999545

[4] Xu XH, Zeng XY, Wang LJ, Liu YN, Liu JM, Qi JL, et al. The disease burden of pancreatic cancer in China in 1990 and 2017. Zhonghua Liu Xing Bing Xue Za Zhi. 2019;40(9):1084–8. 31594150 10.3760/cma.j.issn.0254-6450.2019.09.012

[5] Cai J, Chen H, Lu M, Zhang Y, Lu B, You L, et al. Advances in the epidemiology of pancreatic cancer: Trends, risk factors, screening, and prognosis. Cancer Lett. 2021;520:1–11. 34216688 10.1016/j.canlet.2021.06.027

[6] Silverman DT, Hoover RN, Brown LM, Swanson GM, Schiffman M, Greenberg RS, et al. Why do Black Americans have a higher risk of pancreatic cancer than White Americans? Epidemiology. 2003;14(1):45–54. 12500045 10.1097/00001648-200301000-00013

[7] Munigala S, Subramaniam DS, Subramaniam DP, Burroughs TE, Conwell DL, Sheth SG. Incidence and risk of pancreatic cancer in patients with a new diagnosis of chronic pancreatitis. Dig Dis Sci. 2022;67(2):708–15. 33630214 10.1007/s10620-021-06886-7

[8] Kim HS, Gweon TG, Park SH, Kim TH, Kim CW, Chang JH. Incidence and risk of pancreatic cancer in patients with chronic pancreatitis: defining the optimal subgroup for surveillance. Sci Rep. 2023;13(1):106. 36596818 10.1038/s41598-022-26411-8 PMC9810784

[9] Mellenthin C, Balaban VD, Dugic A, Cullati S. Risk factors for pancreatic cancer in patients with new-onset diabetes: a systematic review and meta-analysis. Cancers (Basel). 2022. 10.3390/cancers14194684. 36230607 10.3390/cancers14194684 PMC9563634

[10] Zhao JF, Teng QP, Lv Y, Li XY, Ding Y. Association between hepatitis B or hepatitis C virus infection and risk of pancreatic cancer: a systematic review and meta-analysis of cohort studies. Ther Adv Infect Dis. 2023;10:20499361231212161. 37954404 10.1177/20499361231212161 PMC10634262

[11] Sawhney MS, Calderwood AH, Thosani NC, Rebbeck TR, Wani S, Canto MI, et al. ASGE guideline on screening for pancreatic cancer in individuals with genetic susceptibility: summary and recommendations. Gastrointest Endosc. 2022;95(5):817–26. 35183358 10.1016/j.gie.2021.12.001

[12] Conroy T, Pfeiffer P, Vilgrain V, Lamarca A, Seufferlein T, O’Reilly EM, et al. Pancreatic cancer: ESMO Clinical Practice Guideline for diagnosis, treatment and follow-up. Ann Oncol. 2023;34(11):987–1002. 37678671 10.1016/j.annonc.2023.08.009

[13] Okusaka T, Nakamura M, Yoshida M, Kitano M, Ito Y, Mizuno N, et al. Clinical Practice Guidelines for Pancreatic Cancer 2022 from the Japan Pancreas Society: a synopsis. Int J Clin Oncol. 2023;28(4):493–511. 36920680 10.1007/s10147-023-02317-x PMC10066137

[14] Goggins M, Overbeek KA, Brand R, Syngal S, Del Chiaro M, Bartsch DK, et al. Management of patients with increased risk for familial pancreatic cancer: updated recommendations from the International Cancer of the Pancreas Screening (CAPS) Consortium. Gut. 2020;69(1):7–17. 31672839 10.1136/gutjnl-2019-319352 PMC7295005

[15] Galli C, Basso D, Plebani M. CA 19-9: handle with care. Clin Chem Lab Med. 2013;51(7):1369–83. 23370912 10.1515/cclm-2012-0744

[16] Sasaki A, Sakata K, Nakano K, Tsutsumi S, Fujishima H, Futsukaichi T, et al. DUPAN-2 as a Risk Factor of Early Recurrence After Curative Pancreatectomy for Patients With Pancreatic Ductal Adenocarcinoma. Pancreas. 2023;52(2):e110–4. 37523601 10.1097/MPA.0000000000002209

[17] Ando Y, Dbouk M, Yoshida T, Saba H, Abou Diwan E, Yoshida K, et al. Using tumor marker gene variants to improve the diagnostic accuracy of DUPAN-2 and carbohydrate antigen 19-9 for pancreatic cancer. J Clin Oncol. 2024;42(18):2196–206. 38457748 10.1200/JCO.23.01573 PMC11191066

[18] Abe T, Koi C, Kohi S, Song KB, Tamura K, Macgregor-Das A, et al. Gene variants that affect levels of circulating tumor markers increase identification of patients with pancreatic cancer. Clin Gastroenterol Hepatol. 2020;18(5):1161-9.e5. 31676359 10.1016/j.cgh.2019.10.036 PMC7166164

[19] Dbouk M, Abe T, Koi C, Ando Y, Saba H, Abou Diwan E, et al. Diagnostic performance of a tumor marker gene test to personalize serum CA19-9 reference ranges. Clin Cancer Res. 2023;29(20):4178–85. 37566230 10.1158/1078-0432.CCR-23-0655 PMC10570677

[20] Kitano M, Yoshida T, Itonaga M, Tamura T, Hatamaru K, Yamashita Y. Impact of endoscopic ultrasonography on diagnosis of pancreatic cancer. J Gastroenterol. 2019;54(1):19–32. 30406288 10.1007/s00535-018-1519-2 PMC6314985

[21] Toft J, Hadden WJ, Laurence JM, Lam V, Yuen L, Janssen A, et al. Imaging modalities in the diagnosis of pancreatic adenocarcinoma: A systematic review and meta-analysis of sensitivity, specificity and diagnostic accuracy. Eur J Radiol. 2017;92:17–23. 28624015 10.1016/j.ejrad.2017.04.009

[22] Vasen HFA, Boekestijn B, Ibrahim IS, Inderson A, Bonsing BA, de Vos Tot Nerveen Cappel WH, et al. Dilatation of the main pancreatic duct as first manifestation of small pancreatic ductal adenocarcinomas detected in a hereditary pancreatic cancer surveillance program. HPB (Oxf). 2019;21(10):1371–5. 10.1016/j.hpb.2019.02.013 30910317

[23] Miura S, Takikawa T, Kikuta K, Hamada S, Kume K, Yoshida N, et al. Focal parenchymal atrophy of the pancreas is frequently observed on pre-diagnostic computed tomography in patients with pancreatic cancer: a case-control study. Diagnostics (Basel). 2021;11(9):1693. 34574034 10.3390/diagnostics11091693 PMC8471718

[24] Sah RP, Sharma A, Nagpal S, Patlolla SH, Sharma A, Kandlakunta H, et al. Phases of metabolic and soft tissue changes in months preceding a diagnosis of pancreatic ductal adenocarcinoma. Gastroenterology. 2019;156(6):1742–52. 30677401 10.1053/j.gastro.2019.01.039 PMC6475474

[25] Danai LV, Babic A, Rosenthal MH, Dennstedt EA, Muir A, Lien EC, et al. Altered exocrine function can drive adipose wasting in early pancreatic cancer. Nature. 2018;558(7711):600–4. 29925948 10.1038/s41586-018-0235-7 PMC6112987

[26] Park MJ, Kim YK, Choi SY, Rhim H, Lee WJ, Choi D. Preoperative detection of small pancreatic carcinoma: value of adding diffusion-weighted imaging to conventional MR imaging for improving confidence level. Radiology. 2014;273(2):433–43. 24991989 10.1148/radiol.14132563

[27] Kim JH, Park SH, Yu ES, Kim MH, Kim J, Byun JH, et al. Visually isoattenuating pancreatic adenocarcinoma at dynamic-enhanced CT: frequency, clinical and pathologic characteristics, and diagnosis at imaging examinations. Radiology. 2010;257(1):87–96. 20697118 10.1148/radiol.10100015

[28] Bilreiro C, Andrade L, Santiago I, Marques RM, Matos C. Imaging of pancreatic ductal adenocarcinoma - an update for all stages of patient management. Eur J Radiol Open. 2024;12:100553. 38357385 10.1016/j.ejro.2024.100553 PMC10864763

[29] Chatterjee A, Shah J. Role of endoscopic ultrasound in diagnosis of pancreatic ductal adenocarcinoma. Diagnostics. 2023. 10.3390/diagnostics14010078. 38201387 10.3390/diagnostics14010078 PMC10802852

[30] Kitano M, Kudo M, Yamao K, Takagi T, Sakamoto H, Komaki T, et al. Characterization of small solid tumors in the pancreas: the value of contrast-enhanced harmonic endoscopic ultrasonography. Am J Gastroenterol. 2012;107(2):303–10. 22008892 10.1038/ajg.2011.354

[31] Lin L, Fan Y, Gao F, Jin L, Li D, Sun W, et al. UTMD-promoted co-delivery of gemcitabine and miR-21 inhibitor by dendrimer-entrapped gold nanoparticles for pancreatic cancer therapy. Theranostics. 2018;8(7):1923–39. 29556365 10.7150/thno.22834 PMC5858509

[32] Shimamoto N, Imazu H, Homma S, Sumiyama K. Vascular endothelial growth factor receptor 2-targeted ultrasound contrast agent selectively accumulates in pancreatic carcinoma in the allograft mouse model: a pilot study using time-intensity curve analysis of EUS imaging. Endosc Ultrasound. 2019;8(1):69–71. 30777942 10.4103/eus.eus_43_18 PMC6400092

[33] Shimamoto N, Ito M, Chiba M, Honma S, Imazu H, Sumiyama K. Antitumor effect of VEGFR2-targeted microbubble destruction with gemcitabine using an endoscopic ultrasound probe: in vivo mouse pancreatic ductal adenocarcinoma model. Hepatobiliary Pancreat Dis Int. 2020;19(5):478–85. 32265136 10.1016/j.hbpd.2020.03.004

[34] Zhao G, Jiang R, Shi Y, Gao S, Wang D, Li Z, et al. Circulating cell-free DNA methylation-based multi-omics analysis allows early diagnosis of pancreatic ductal adenocarcinoma. Mol Oncol. 2024;18(11):2801–13. 38561976 10.1002/1878-0261.13643 PMC11547243

[35] Wu J, Xu X, Zhang Q, Li P, Wu T, Guo S, et al. Cell-free DNA testing for the detection and prognosis prediction of pancreatic cancer. Nat Commun. 2025;16(1):6645. 40681511 10.1038/s41467-025-61890-z PMC12274360

[36] Macgregor-Das A, Yu J, Tamura K, Abe T, Suenaga M, Shindo K, et al. Detection of circulating tumor DNA in patients with pancreatic cancer using digital next-generation sequencing. J Mol Diagn. 2020;22(6):748–56. 32205290 10.1016/j.jmoldx.2020.02.010 PMC7338889

[37] Evrard C, Ingrand P, Rochelle T, Martel M, Tachon G, Flores N, et al. Circulating tumor DNA in unresectable pancreatic cancer is a strong predictor of first-line treatment efficacy: the KRASCIPANC prospective study. Dig Liver Dis. 2023;55(11):1562–72. 37308396 10.1016/j.dld.2023.03.011

[38] Steiniche MM, Lindgaard SC, Chen IM, Johansen JS, Andersen RF, Hansen TF, et al. The prognostic impact of early ctDNA kinetics in metastatic pancreatic cancer using the ctDNA Response Evaluation Criteria in Solid Tumors (ctDNA-RECIST). Clin Cancer Res. 2025;31:4720–9. 40970773 10.1158/1078-0432.CCR-25-0758 PMC12616238

[39] Peng C, Wang J, Gao W, Huang L, Liu Y, Li X, et al. Meta-analysis of the diagnostic performance of circulating MicroRNAs for pancreatic cancer. Int J Med Sci. 2021;18(3):660–71. 33437201 10.7150/ijms.52706 PMC7797557

[40] Wang C, Cai H, Cai Q, Wu J, Stolzenberg-Solomon R, Guo X, et al. Circulating microRNAs in association with pancreatic cancer risk within 5 years. Int J Cancer. 2024;155(3):519–31. 38602070 10.1002/ijc.34956 PMC11214275

[41] Hartlapp I, Valta-Seufzer D, Siveke JT, Algül H, Goekkurt E, Siegler G, et al. Prognostic and predictive value of CA 19-9 in locally advanced pancreatic cancer treated with multiagent induction chemotherapy: results from a prospective, multicenter phase II trial (NEOLAP-AIO-PAK-0113). ESMO Open. 2022;7(4):100552. 35970013 10.1016/j.esmoop.2022.100552 PMC9434418

[42] Ferrone CR, Finkelstein DM, Thayer SP, Muzikansky A, Fernandez-delCastillo C, Warshaw AL. Perioperative CA19-9 levels can predict stage and survival in patients with resectable pancreatic adenocarcinoma. J Clin Oncol. 2006;24(18):2897–902. 16782929 10.1200/JCO.2005.05.3934 PMC3817569

[43] Chuong MD, Boggs DH, Patel KN, Regine WF. Adjuvant chemoradiation for pancreatic cancer: what does the evidence tell us? J Gastrointest Oncol. 2014;5(3):166–77. 24982765 10.3978/j.issn.2078-6891.2014.025 PMC4074954

[44] Kim J, Bamlet WR, Oberg AL, Chaffee KG, Donahue G, Cao XJ, et al. Detection of early pancreatic ductal adenocarcinoma with thrombospondin-2 and CA19-9 blood markers. Sci Transl Med. 2017. 10.1126/scitranslmed.aah5583. 28701476 10.1126/scitranslmed.aah5583 PMC5727893

[45] Fahrmann JF, Bantis LE, Capello M, Scelo G, Dennison JB, Patel N, et al. A plasma-derived protein-metabolite multiplexed panel for early-stage pancreatic cancer. J Natl Cancer Inst. 2019;111(4):372–9. 30137376 10.1093/jnci/djy126 PMC6449169

[46] Brand RE, Persson J, Bratlie SO, Chung DC, Katona BW, Carrato A, et al. Detection of early-stage pancreatic ductal adenocarcinoma from blood samples: results of a multiplex biomarker signature validation study. Clin Transl Gastroenterol. 2022;13(3):e00468. 35166713 10.14309/ctg.0000000000000468 PMC8963856

[47] King TCM. The IMMray PanCan-d Test Performance and CA19–9: results from the blind validation study. Am J Gastroenterol. 2021;S8(116):S4.

[48] Katona BW, Worthington C, Clay D, Cincotta H, Ahmad NA, Ginsberg GG, et al. Outcomes of the IMMray PanCan-d test in high-risk individuals undergoing pancreatic surveillance: pragmatic data and lessons learned. JCO Precis Oncol. 2023;7:e2300445. 37883920 10.1200/PO.23.00445

[49] Debernardi S, Blyuss O, Rycyk D, Srivastava K, Jeon CY, Cai H, et al. Urine biomarkers enable pancreatic cancer detection up to 2 years before diagnosis. Int J Cancer. 2023;152(4):769–80. 36093581 10.1002/ijc.34287 PMC9789171

[50] Debernardi S, O’Brien H, Algahmdi AS, Malats N, Stewart GD, Plješa-Ercegovac M, et al. A combination of urinary biomarker panel and PancRISK score for earlier detection of pancreatic cancer: A case-control study. PLoS Med. 2020;17(12):e1003489. 33301466 10.1371/journal.pmed.1003489 PMC7758047

[51] Hogendorf P, Durczyński A, Skulimowski A, Kumor A, Poznańska G, Strzelczyk J. Neutrophil gelatinase-associated lipocalin (NGAL) concentration in urine is superior to CA19-9 and Ca 125 in differentiation of pancreatic mass: preliminary report. Cancer Biomark. 2016;16(4):537–43. 27002756 10.3233/CBM-160595 PMC13016520

[52] Sahni S, Pandya AR, Hadden WJ, Nahm CB, Maloney S, Cook V, et al. A unique urinary metabolomic signature for the detection of pancreatic ductal adenocarcinoma. Int J Cancer. 2021;148(6):1508–18. 33128797 10.1002/ijc.33368

[53] Zhang L, Farrell JJ, Zhou H, Elashoff D, Akin D, Park NH, et al. Salivary transcriptomic biomarkers for detection of resectable pancreatic cancer. Gastroenterology. 2010;138(3):949–57. 19931263 10.1053/j.gastro.2009.11.010 PMC2831159

[54] Xie Z, Yin X, Gong B, Nie W, Wu B, Zhang X, et al. Salivary microRNAs show potential as a noninvasive biomarker for detecting resectable pancreatic cancer. Cancer Prev Res (Phila). 2015;8(2):165–73. 25538087 10.1158/1940-6207.CAPR-14-0192

[55] Al Balushi H, Chowdhury P, Babu HM, Rehman A, Bokhari SFH, Al-Tarawneh LM, et al. The potential of salivary biomarkers in early detection of pancreatic ductal adenocarcinoma: a systematic review. Cureus. 2024;16(2):e55003. 38550499 10.7759/cureus.55003 PMC10973743

[56] Koopaie M, Kolahdooz S, Fatahzadeh M, Aleedawi ZA. Salivary noncoding RNA in the diagnosis of pancreatic cancer: systematic review and meta-analysis. Eur J Clin Invest. 2022;52(12):e13848. 35906804 10.1111/eci.13848

[57] Kim HI, Park J, Zhu Y, Wang X, Han Y, Zhang D. Recent advances in extracellular vesicles for therapeutic cargo delivery. Exp Mol Med. 2024;56(4):836–49. 38556545 10.1038/s12276-024-01201-6 PMC11059217

[58] Hánělová K, Raudenská M, Masařík M, Balvan J. Protein cargo in extracellular vesicles as the key mediator in the progression of cancer. Cell Commun Signal. 2024;22(1):25. 38200509 10.1186/s12964-023-01408-6 PMC10777590

[59] Servage KA, Stefanius K, Gray HF, Orth K. Proteomic profiling of small extracellular vesicles secreted by human pancreatic cancer cells implicated in cellular transformation. Sci Rep. 2020;10(1):7713. 32382024 10.1038/s41598-020-64718-6 PMC7205864

[60] Kren N, Michaud D, Bagchi S, Greene K, Pylayeva-Gupta Y. Rab27a plays a dual role in metastatic propensity of pancreatic cancer. Sci Rep. 2020;10(1):7390. 32355248 10.1038/s41598-020-64248-1 PMC7193593

[61] Hoshino A, Costa-Silva B, Shen TL, Rodrigues G, Hashimoto A, Tesic Mark M, et al. Tumour exosome integrins determine organotropic metastasis. Nature. 2015;527(7578):329–35. 26524530 10.1038/nature15756 PMC4788391

[62] Costa-Silva B, Aiello NM, Ocean AJ, Singh S, Zhang H, Thakur BK, et al. Pancreatic cancer exosomes initiate pre-metastatic niche formation in the liver. Nat Cell Biol. 2015;17(6):816–26. 25985394 10.1038/ncb3169 PMC5769922

[63] Villarroya-Beltri C, Gutiérrez-Vázquez C, Sánchez-Cabo F, Pérez-Hernández D, Vázquez J, Martin-Cofreces N, et al. Sumoylated hnRNPA2B1 controls the sorting of miRNAs into exosomes through binding to specific motifs. Nat Commun. 2013;4:2980. 24356509 10.1038/ncomms3980 PMC3905700

[64] Shurtleff MJ, Temoche-Diaz MM, Karfilis KV, Ri S, Schekman R. Y-box protein 1 is required to sort microRNAs into exosomes in cells and in a cell-free reaction. Elife. 2016. 10.7554/eLife.19276. 27559612 10.7554/eLife.19276 PMC5047747

[65] Santangelo L, Giurato G, Cicchini C, Montaldo C, Mancone C, Tarallo R, et al. The RNA-binding protein SYNCRIP is a component of the hepatocyte exosomal machinery controlling microRNA sorting. Cell Rep. 2016;17(3):799–808. 27732855 10.1016/j.celrep.2016.09.031

[66] Luo Y, Li Z, Kong Y, He W, Zheng H, An M, et al. *KRAS* mutant-driven SUMOylation controls extracellular vesicle transmission to trigger lymphangiogenesis in pancreatic cancer. J Clin Invest. 2022. 10.1172/JCI157644. 35579947 10.1172/JCI157644 PMC9282935

[67] Wang X, Luo G, Zhang K, Cao J, Huang C, Jiang T, et al. Hypoxic tumor-derived exosomal miR-301a mediates M2 macrophage polarization via PTEN/PI3Kγ to promote pancreatic cancer metastasis. Cancer Res. 2018;78(16):4586–98. 29880482 10.1158/0008-5472.CAN-17-3841

[68] Mulcahy LA, Pink RC, Carter DR. Routes and mechanisms of extracellular vesicle uptake. J Extracell Vesicles. 2014. 10.3402/jev.v3.24641. 25143819 10.3402/jev.v3.24641 PMC4122821

[69] Song S, Zhang Y, Ding T, Ji N, Zhao H. The dual role of macropinocytosis in cancers: promoting growth and inducing methuosis to participate in anticancer therapies as targets. Front Oncol. 2020;10:570108. 33542897 10.3389/fonc.2020.570108 PMC7851083

[70] Xiao F, Li J, Huang K, Li X, Xiong Y, Wu M, et al. Macropinocytosis: mechanism and targeted therapy in cancers. Am J Cancer Res. 2021;11(1):14–30. 33520357 PMC7840718

[71] Commisso C, Davidson SM, Soydaner-Azeloglu RG, Parker SJ, Kamphorst JJ, Hackett S, et al. Macropinocytosis of protein is an amino acid supply route in Ras-transformed cells. Nature. 2013;497(7451):633–7. 23665962 10.1038/nature12138 PMC3810415

[72] Kamerkar S, LeBleu VS, Sugimoto H, Yang S, Ruivo CF, Melo SA, et al. Exosomes facilitate therapeutic targeting of oncogenic KRAS in pancreatic cancer. Nature. 2017;546(7659):498–503. 28607485 10.1038/nature22341 PMC5538883

[73] Lan B, Zeng S, Grützmann R, Pilarsky C. The role of exosomes in pancreatic cancer. Int J Mol Sci. 2019. 10.3390/ijms20184332. 31487880 10.3390/ijms20184332 PMC6770781

[74] Lorenzon L, Blandino G. Glypican-1 exosomes: do they initiate a new era for early pancreatic cancer diagnosis? Transl Gastroenterol Hepatol. 2016;1:8. 28164166 10.21037/tgh.2016.01.07 PMC5289289

[75] Yu Z, Zhao S, Ren L, Wang L, Chen Z, Hoffman RM, et al. Pancreatic cancer-derived exosomes promote tumor metastasis and liver pre-metastatic niche formation. Oncotarget. 2017;8(38):63461–83. 28969005 10.18632/oncotarget.18831 PMC5609937

[76] Zhao Y, Feng Y, Sun F, Li L, Chen J, Song Y, et al. Optimized rAAV8 targeting acinar KLF4 ameliorates fibrosis in chronic pancreatitis via exosomes-enriched let-7s suppressing pancreatic stellate cells activation. Mol Ther. 2024;32(8):2624–40. 38956871 10.1016/j.ymthe.2024.06.030 PMC11405174

[77] Adem B, Bastos N, Ruivo CF, Sousa-Alves S, Dias C, Vieira PF, et al. Exosomes define a local and systemic communication network in healthy pancreas and pancreatic ductal adenocarcinoma. Nat Commun. 2024;15(1):1496. 38383468 10.1038/s41467-024-45753-7 PMC10881969

[78] Chen K, Wang Q, Liu X, Wang F, Yang Y, Tian X. Hypoxic pancreatic cancer derived exosomal miR-30b-5p promotes tumor angiogenesis by inhibiting GJA1 expression. Int J Biol Sci. 2022;18(3):1220–37. 35173549 10.7150/ijbs.67675 PMC8771853

[79] Wu G, Ding X, Quan G, Xiong J, Li Q, Li Z, et al. Hypoxia-induced miR-210 promotes endothelial cell permeability and angiogenesis via exosomes in pancreatic ductal adenocarcinoma. Biochem Res Int. 2022;2022:7752277. 36466111 10.1155/2022/7752277 PMC9718630

[80] Shang D, Xie C, Hu J, Tan J, Yuan Y, Liu Z, et al. Pancreatic cancer cell-derived exosomal microRNA-27a promotes angiogenesis of human microvascular endothelial cells in pancreatic cancer via BTG2. J Cell Mol Med. 2020;24(1):588–604. 31724333 10.1111/jcmm.14766 PMC6933412

[81] Wang T, Ye L, Zhou Y, Zhang X, Li R, Zhou Y, et al. Pancreatic cancer-derived exosomal miR-510 promotes macrophage M2 polarization and facilitates cancer cell aggressive phenotypes. Hum Cell. 2024;38(1):17. 39528705 10.1007/s13577-024-01144-0

[82] Dai E, Han L, Liu J, Xie Y, Kroemer G, Klionsky DJ, et al. Autophagy-dependent ferroptosis drives tumor-associated macrophage polarization via release and uptake of oncogenic KRAS protein. Autophagy. 2020;16(11):2069–83. 31920150 10.1080/15548627.2020.1714209 PMC7595620

[83] Zhao M, Zhuang A, Fang Y. Cancer-associated fibroblast-derived exosomal miRNA-320a promotes macrophage M2 polarization in vitro by regulating PTEN/PI3Kγ signaling in pancreatic cancer. J Oncol. 2022;2022:9514697. 35813857 10.1155/2022/9514697 PMC9270150

[84] Javeed N, Sagar G, Dutta SK, Smyrk TC, Lau JS, Bhattacharya S, et al. Pancreatic cancer-derived exosomes cause paraneoplastic β-cell dysfunction. Clin Cancer Res. 2015;21(7):1722–33. 25355928 10.1158/1078-0432.CCR-14-2022 PMC4383684

[85] Pang W, Yao W, Dai X, Zhang A, Hou L, Wang L, et al. Pancreatic cancer-derived exosomal microRNA-19a induces β-cell dysfunction by targeting ADCY1 and EPAC2. Int J Biol Sci. 2021;17(13):3622–33. 34512170 10.7150/ijbs.56271 PMC8416731

[86] Su J, Pang W, Zhang A, Li L, Yao W, Dai X. Exosomal miR-19a decreases insulin production by targeting Neurod1 in pancreatic cancer associated diabetes. Mol Biol Rep. 2022;49(3):1711–20. 34854011 10.1007/s11033-021-06980-z

[87] Liu C, He D, Li L, Zhang S, Wang L, Fan Z, et al. Extracellular vesicles in pancreatic cancer immune escape: emerging roles and mechanisms. Pharmacol Res. 2022;183:106364. 35901939 10.1016/j.phrs.2022.106364

[88] Ormseth B, Onuma A, Zhang H, Tsung A. The hepatic pre-metastatic niche. Cancers (Basel). 2022. 10.3390/cancers14153731. 35954395 10.3390/cancers14153731 PMC9367402

[89] Patel GK, Khan MA, Bhardwaj A, Srivastava SK, Zubair H, Patton MC, et al. Exosomes confer chemoresistance to pancreatic cancer cells by promoting ROS detoxification and miR-155-mediated suppression of key gemcitabine-metabolising enzyme. DCK Br J Cancer. 2017;116(5):609–19. 28152544 10.1038/bjc.2017.18 PMC5344296

[90] Pan Y, Tang H, Li Q, Chen G, Li D. Exosomes and their roles in the chemoresistance of pancreatic cancer. Cancer Med. 2022;11(24):4979–88. 35587712 10.1002/cam4.4830 PMC9761084

[91] Mikamori M, Yamada D, Eguchi H, Hasegawa S, Kishimoto T, Tomimaru Y, et al. MicroRNA-155 controls exosome synthesis and promotes gemcitabine resistance in pancreatic ductal adenocarcinoma. Sci Rep. 2017;7:42339. 28198398 10.1038/srep42339 PMC5309735

[92] Li X, Li K, Li M, Lin X, Mei Y, Huang X, et al. Chemoresistance transmission via exosome-transferred MMP14 in pancreatic cancer. Front Oncol. 2022;12:844648. 35223528 10.3389/fonc.2022.844648 PMC8865617

[93] Yang Z, Zhao N, Cui J, Wu H, Xiong J, Peng T. Exosomes derived from cancer stem cells of gemcitabine-resistant pancreatic cancer cells enhance drug resistance by delivering miR-210. Cell Oncol (Dordr). 2020;43(1):123–36. 31713003 10.1007/s13402-019-00476-6 PMC12990725

[94] Melo SA, Luecke LB, Kahlert C, Fernandez AF, Gammon ST, Kaye J, et al. Glypican-1 identifies cancer exosomes and detects early pancreatic cancer. Nature. 2015;523(7559):177–82. 26106858 10.1038/nature14581 PMC4825698

[95] Qiao Z, Wang E, Bao B, Tan X, Chen H, Wang D, et al. Diagnostic and prognostic value of circulating exosomal glypican-1 in pancreatic cancer: a meta-analysis. Lab Med. 2024;55(5):543–52. 38470244 10.1093/labmed/lmae013

[96] Frampton AE, Prado MM, López-Jiménez E, Fajardo-Puerta AB, Jawad ZAR, Lawton P, et al. Glypican-1 is enriched in circulating-exosomes in pancreatic cancer and correlates with tumor burden. Oncotarget. 2018;9(27):19006–13. 29721179 10.18632/oncotarget.24873 PMC5922373

[97] Jia E, Ren N, Shi X, Zhang R, Yu H, Yu F, et al. Extracellular vesicle biomarkers for pancreatic cancer diagnosis: a systematic review and meta-analysis. BMC Cancer. 2022;22(1):573. 35606727 10.1186/s12885-022-09463-x PMC9125932

[98] Yu H, Xin C, Zhou Y, Ding X. Advances in the application of extracellular vesicles in precise diagnosis of pancreatic cancer. Eur J Med Res. 2025;30(1):478. 40514731 10.1186/s40001-025-02739-5 PMC12164127

[99] Xiao D, Dong Z, Zhen L, Xia G, Huang X, Wang T, et al. Combined exosomal GPC1, CD82, and serum CA19-9 as multiplex targets: a specific, sensitive, and reproducible detection panel for the diagnosis of pancreatic cancer. Mol Cancer Res. 2020;18(2):300–10. 31662449 10.1158/1541-7786.MCR-19-0588

[100] Li H, Chiang CL, Kwak KJ, Wang X, Doddi S, Ramanathan LV, et al. Extracellular vesicular analysis of Glypican 1 mRNA and protein for pancreatic cancer diagnosis and prognosis. Adv Sci (Weinh). 2024;11(11):e2306373. 38204202 10.1002/advs.202306373 PMC10953589

[101] Toracchio L, Carrabotta M, Mancarella C, Morrione A, Scotlandi K. EphA2 in cancer: molecular complexity and therapeutic opportunities. Int J Mol Sci. 2024. 10.3390/ijms252212191. 39596256 10.3390/ijms252212191 PMC11594831

[102] Wei Q, Zhang J, Li Z, Wei L, Ren L. Serum Exo-EphA2 as a potential diagnostic biomarker for pancreatic cancer. Pancreas. 2020;49(9):1213–9. 32898008 10.1097/MPA.0000000000001660

[103] Wei Q, Li Z, Feng H, Ren L. Serum exosomal EphA2 is a prognostic biomarker in patients with pancreatic cancer. Cancer Manag Res. 2021;13:3675–83. 33994808 10.2147/CMAR.S304719 PMC8112875

[104] Fan J, Wei Q, Koay EJ, Liu Y, Ning B, Bernard PW, et al. Chemoresistance transmission via exosome-mediated EphA2 transfer in pancreatic cancer. Theranostics. 2018;8(21):5986–94. 30613276 10.7150/thno.26650 PMC6299429

[105] Tiwari PK, Shanmugam P, Karn V, Gupta S, Mishra R, Rustagi S, et al. Extracellular vesicular miRNA in pancreatic cancer: from lab to therapy. Cancers (Basel). 2024. 10.3390/cancers16122179. 38927885 10.3390/cancers16122179 PMC11201547

[106] Lai X, Wang M, McElyea SD, Sherman S, House M, Korc M. A microRNA signature in circulating exosomes is superior to exosomal glypican-1 levels for diagnosing pancreatic cancer. Cancer Lett. 2017;393:86–93. 28232049 10.1016/j.canlet.2017.02.019 PMC5386003

[107] Nakamura K, Zhu Z, Roy S, Jun E, Han H, Munoz RM, et al. An exosome-based transcriptomic signature for noninvasive, early detection of patients with pancreatic ductal adenocarcinoma: a multicenter Cohort Study. Gastroenterology. 2022;163(5):1252-66.e2. 35850192 10.1053/j.gastro.2022.06.090 PMC9613527

[108] Xie Z, Chen X, Li J, Guo Y, Li H, Pan X, et al. Salivary HOTAIR and PVT1 as novel biomarkers for early pancreatic cancer. Oncotarget. 2016;7(18):25408–19. 27028998 10.18632/oncotarget.8323 PMC5041913

[109] Kumar SR, Kimchi ET, Manjunath Y, Gajagowni S, Stuckel AJ, Kaifi JT. RNA cargos in extracellular vesicles derived from blood serum in pancreas associated conditions. Sci Rep. 2020;10(1):2800. 32071328 10.1038/s41598-020-59523-0 PMC7028741

[110] Takahashi K, Ota Y, Kogure T, Suzuki Y, Iwamoto H, Yamakita K, et al. Circulating extracellular vesicle-encapsulated HULC is a potential biomarker for human pancreatic cancer. Cancer Sci. 2020;111(1):98–111. 31715081 10.1111/cas.14232 PMC6942436

[111] He X, Chen L, Di Y, Li W, Zhang X, Bai Z, et al. Plasma-derived exosomal long noncoding RNAs of pancreatic cancer patients as novel blood-based biomarkers of disease. BMC Cancer. 2024;24(1):961. 39107726 10.1186/s12885-024-12755-z PMC11301836

[112] Liang X, Qi M, Wu R, Liu A, Chen D, Tang L, et al. Long non-coding RNA CUDR promotes malignant phenotypes in pancreatic ductal adenocarcinoma via activating AKT and ERK signaling pathways. Int J Oncol. 2018;53(6):2671–82. 30272271 10.3892/ijo.2018.4574

[113] Fang X, Cai Y, Xu Y, Zhang H. Exosome-mediated lncRNA SNHG11 regulates angiogenesis in pancreatic carcinoma through miR-324-3p/VEGFA axis. Cell Biol Int. 2022;46(1):106–17. 34519129 10.1002/cbin.11703

[114] Yao J, Gao R, Luo M, Li D, Guo L, Yu Z, et al. Exosomal LINC00460/miR-503-5p/ANLN positive feedback loop aggravates pancreatic cancer progression through regulating T cell-mediated cytotoxicity and PD-1 checkpoint. Cancer Cell Int. 2022;22(1):390. 36482354 10.1186/s12935-022-02741-5 PMC9733079

[115] Li Z, Jiang P, Li J, Peng M, Zhao X, Zhang X, et al. Tumor-derived exosomal lnc-Sox2ot promotes EMT and stemness by acting as a ceRNA in pancreatic ductal adenocarcinoma. Oncogene. 2018;37(28):3822–38. 29643475 10.1038/s41388-018-0237-9

[116] Wang H, Min J, Xu C, Liu Y, Yu Z, Gong A, et al. Hypoxia-elicited exosomes promote the chemoresistance of pancreatic cancer cells by transferring LncROR via Hippo signaling. J Cancer. 2023;14(6):1075–87. 37151398 10.7150/jca.81320 PMC10158512

[117] Kahlert C, Melo SA, Protopopov A, Tang J, Seth S, Koch M, et al. Identification of double-stranded genomic DNA spanning all chromosomes with mutated KRAS and p53 DNA in the serum exosomes of patients with pancreatic cancer. J Biol Chem. 2014;289(7):3869–75. 24398677 10.1074/jbc.C113.532267 PMC3924256

[118] Yang S, Che SP, Kurywchak P, Tavormina JL, Gansmo LB, de Correa Sampaio P, et al. Detection of mutant *KRAS* and TP53 DNA in circulating exosomes from healthy individuals and patients with pancreatic cancer. Cancer Biol Ther. 2017;18(3):158–65. 28121262 10.1080/15384047.2017.1281499 PMC5389423

[119] Allenson K, Castillo J, San Lucas FA, Scelo G, Kim DU, Bernard V, et al. High prevalence of mutant KRAS in circulating exosome-derived DNA from early-stage pancreatic cancer patients. Ann Oncol. 2017;28(4):741–7. 28104621 10.1093/annonc/mdx004 PMC5834026

[120] Bernard V, Kim DU, San Lucas FA, Castillo J, Allenson K, Mulu FC, et al. Circulating nucleic acids are associated with outcomes of patients with pancreatic cancer. Gastroenterology. 2019;156(1):108-18.e4. 30240661 10.1053/j.gastro.2018.09.022 PMC6434712

[121] Hinestrosa JP, Sears RC, Dhani H, Lewis JM, Schroeder G, Balcer HI, et al. Development of a blood-based extracellular vesicle classifier for detection of early-stage pancreatic ductal adenocarcinoma. Commun Med (Lond). 2023;3(1):146. 37857666 10.1038/s43856-023-00351-4 PMC10587093

[122] Bockorny B, Muthuswamy L, Huang L, Hadisurya M, Maria Lim C, Tsai LL, et al. A large-scale proteomics resource of circulating extracellular vesicles for biomarker discovery in pancreatic cancer. Elife. 2024;12:RP87369. 39693144 10.7554/eLife.87369 PMC11655061

[123] Yoshioka Y, Kosaka N, Konishi Y, Ohta H, Okamoto H, Sonoda H, et al. Ultra-sensitive liquid biopsy of circulating extracellular vesicles using exoscreen. Nat Commun. 2014;5:3591. 24710016 10.1038/ncomms4591 PMC3988821

[124] Lewis JM, Vyas AD, Qiu Y, Messer KS, White R, Heller MJ. Integrated analysis of exosomal protein biomarkers on alternating current electrokinetic chips enables rapid detection of pancreatic cancer in patient blood. ACS Nano. 2018;12(4):3311–20. 29570265 10.1021/acsnano.7b08199

[125] Torasawa M, Horinouchi H, Yagishita S, Utsumi H, Okuda K, Takekoshi D, et al. Exploratory analysis to predict pneumonitis during durvalumab consolidation therapy for patients with locally advanced non-small cell lung cancer from proteomic profiling of circulating extracellular vesicles. Thorac Cancer. 2023;14(29):2909–23. 37614219 10.1111/1759-7714.15077 PMC10569905

[126] Utsumi H, Yagishita S, Kawajiri K, Torasawa M, Kiritani A, Tamura K, et al. Identification of poor prognostic factors using circulating extracellular vesicles in durvalumab consolidation therapy for locally advanced non-small cell lung cancer. Lung Cancer. 2025;208:108732. 40925068 10.1016/j.lungcan.2025.108732

[127] Takahashi K, Inuzuka T, Shimizu Y, Sawamoto K, Taniue K, Ono Y, et al. Liquid biopsy for pancreatic cancer by serum extracellular vesicle-encapsulated long noncoding RNA HEVEPA. Pancreas. 2024;53(5):e395–404. 38416857 10.1097/MPA.0000000000002315

[128] Pantel K, Alix-Panabières C, Hofman P, Stoecklein NH, Lu YJ, Lianidou E, et al. Fostering the implementation of liquid biopsy in clinical practice: meeting report 2024 of the European Liquid Biopsy Society (ELBS). J Exp Clin Cancer Res. 2025;44(1):156. 40410806 10.1186/s13046-025-03398-4 PMC12100835

[129] Lockwood CM, Merker JD, Bain E, Compton C, Grossman RL, Johann D, et al. Towards preanalytical best practices for liquid biopsy studies: a BLOODPAC landscape analysis. Clin Pharmacol Ther. 2025;117(1):28–33. 39164947 10.1002/cpt.3416 PMC11652819

[130] Connors D, Allen J, Alvarez JD, Boyle J, Cristofanilli M, Hiller C, et al. International liquid biopsy standardization alliance white paper. Crit Rev Oncol Hematol. 2020;156:103112. 33035734 10.1016/j.critrevonc.2020.103112

[131] H.U.中央研究所が「膵がん早期診断コンソーシアム」を発足 2025.

[132] Ferguson S, Yang KS, Weissleder R. Single extracellular vesicle analysis for early cancer detection. Trends Mol Med. 2022;28(8):681–92. 35624008 10.1016/j.molmed.2022.05.003 PMC9339504

[133] Qiu L, Liu X, Zhu L, Luo L, Sun N, Pei R. Current advances in technologies for single extracellular vesicle analysis and its clinical applications in cancer diagnosis. Biosensors. 2023. 10.3390/bios13010129. 36671964 10.3390/bios13010129 PMC9856491

[134] Ferguson S, Yang KS, Zelga P, Liss AS, Carlson JCT, Del Castillo CF, et al. Single-EV analysis (sEVA) of mutated proteins allows detection of stage 1 pancreatic cancer. Sci Adv. 2022;8(16):eabm3453. 35452280 10.1126/sciadv.abm3453 PMC9032977

[135] Yang J, Zhang Y, Gao X, Yuan Y, Zhao J, Zhou S, et al. Plasma-derived exosomal ALIX as a novel biomarker for diagnosis and classification of pancreatic cancer. Front Oncol. 2021;11:628346. 34026608 10.3389/fonc.2021.628346 PMC8131866

[136] Lennon KM, Wakefield DL, Maddox AL, Brehove MS, Willner AN, Garcia-Mansfield K, et al. Single molecule characterization of individual extracellular vesicles from pancreatic cancer. J Extracell Vesicles. 2019;8(1):1685634. 31741725 10.1080/20013078.2019.1685634 PMC6844376

[137] Shin H, Choi BH, Shim O, Kim J, Park Y, Cho SK, et al. Single test-based diagnosis of multiple cancer types using Exosome-SERS-AI for early stage cancers. Nat Commun. 2023;14(1):1644. 36964142 10.1038/s41467-023-37403-1 PMC10039041

[138] Yu J, Sadakari Y, Shindo K, Suenaga M, Brant A, Almario JAN, et al. Digital next-generation sequencing identifies low-abundance mutations in pancreatic juice samples collected from the duodenum of patients with pancreatic cancer and intraductal papillary mucinous neoplasms. Gut. 2017;66(9):1677–87. 27432539 10.1136/gutjnl-2015-311166 PMC5243915

[139] Satoh K. Molecular approaches using body fluid for the early detection of pancreatic cancer. Diagnostics. 2021. 10.3390/diagnostics11020375. 33671729 10.3390/diagnostics11020375 PMC7926932

[140] Suenaga M, Dudley B, Karloski E, Borges M, Irene Canto M, Brand RE, et al. The effect of pancreatic juice collection time on the detection of KRAS mutations. Pancreas. 2018;47(1):35–9. 29200129 10.1097/MPA.0000000000000956 PMC6435266

[141] Li Z, Liu Y, Fu J, Mugaanyi J, Yan J, Lu C, et al. Bile is a reliable and valuable source to study cfDNA in biliary tract cancers. Front Oncol. 2022;12:961939. 36091112 10.3389/fonc.2022.961939 PMC9459008

[142] Driescher C, Fuchs K, Haeberle L, Goering W, Frohn L, Opitz FV, et al. Bile-based cell-free DNA analysis is a reliable diagnostic tool in pancreatobiliary cancer. Cancers (Basel). 2020. 10.3390/cancers13010039. 33375555 10.3390/cancers13010039 PMC7818177

[143] Arechederra M, Rullán M, Amat I, Oyon D, Zabalza L, Elizalde M, et al. Next-generation sequencing of bile cell-free DNA for the early detection of patients with malignant biliary strictures. Gut. 2022;71(6):1141–51. 34285068 10.1136/gutjnl-2021-325178 PMC9120390

[144] Yachida S, Yoshinaga S, Shiba S, Urabe M, Tanaka H, Takeda Y, et al. KRAS mutations in duodenal lavage fluid after secretin stimulation for detection of pancreatic cancer. Ann Surg. 2025. 10.1097/SLA.0000000000006645. 39902566 10.1097/SLA.0000000000006645 PMC13557476

[145] Taniguchi T, Ideno N, Araki T, Miura S, Yamamoto M, Nakafusa T, et al. MicroRNA-20a in extracellular vesicles derived from duodenal fluid is a possible biomarker for pancreatic ductal adenocarcinoma. DEN Open. 2024;4(1):e333. 38434144 10.1002/deo2.333 PMC10908371

[146] Catenacci DV, Chapman CG, Xu P, Koons A, Konda VJ, Siddiqui UD, et al. Acquisition of portal venous circulating tumor cells from patients with pancreaticobiliary cancers by endoscopic ultrasound. Gastroenterology. 2015;149(7):1794-803.e4. 26341722 10.1053/j.gastro.2015.08.050 PMC4985007

[147] Heredia-Soto V, Rodríguez-Salas N, Feliu J. Liquid biopsy in pancreatic cancer: are we ready to apply it in the clinical practice? Cancers (Basel). 2021. 10.3390/cancers13081986. 33924143 10.3390/cancers13081986 PMC8074327

[148] Wu G, Xu X, Zhang H, Peng L, Liu C. Liquid biopsy in biliary tract cancers: early diagnosis, precision therapy, and prognostic evaluation. Front Oncol. 2025;15:1705162. 41487574 10.3389/fonc.2025.1705162 PMC12757225

[149] Tavano F, Latiano A, Palmieri O, Gioffreda D, Latiano T, Gentile A, et al. Duodenal fluid analysis as a rewarding approach to detect low-abundance mutations in biliopancreatic cancers. Int J Mol Sci. 2024. 10.3390/ijms25158436. 39126005 10.3390/ijms25158436 PMC11312909

